# Do Vascular Networks Branch Optimally or Randomly across Spatial Scales?

**DOI:** 10.1371/journal.pcbi.1005223

**Published:** 2016-11-30

**Authors:** Elif Tekin, David Hunt, Mitchell G. Newberry, Van M. Savage

**Affiliations:** 1 Department of Biomathematics, University of California, Los Angeles, David Geffen School of Medicine, Los Angeles, California, United States of America; 2 Department of Biology, University of Pennsylvania, Philadelphia, Pennsylvania, United States of America; 3 Department of Ecology and Evolutionary Biology, University of California, Los Angeles, Los Angeles, California, United States of America; 4 Santa Fe Institute, Santa Fe, New Mexico, United States of America; Johns Hopkins University, UNITED STATES

## Abstract

Modern models that derive allometric relationships between metabolic rate and body mass are based on the architectural design of the cardiovascular system and presume sibling vessels are symmetric in terms of radius, length, flow rate, and pressure. Here, we study the cardiovascular structure of the human head and torso and of a mouse lung based on three-dimensional images processed via our software Angicart. In contrast to modern allometric theories, we find systematic patterns of asymmetry in vascular branching, potentially explaining previously documented mismatches between predictions (power-law or concave curvature) and observed empirical data (convex curvature) for the allometric scaling of metabolic rate. To examine why these systematic asymmetries in vascular branching might arise, we construct a mathematical framework to derive predictions based on local, junction-level optimality principles that have been proposed to be favored in the course of natural selection and development. The two most commonly used principles are material-cost optimizations (construction materials or blood volume) and optimization of efficient flow via minimization of power loss. We show that material-cost optimization solutions match with distributions for asymmetric branching across the whole network but do not match well for individual junctions. Consequently, we also explore random branching that is constrained at scales that range from local (junction-level) to global (whole network). We find that material-cost optimizations are the strongest predictor of vascular branching in the human head and torso, whereas locally or intermediately constrained random branching is comparable to material-cost optimizations for the mouse lung. These differences could be attributable to developmentally-programmed local branching for larger vessels and constrained random branching for smaller vessels.

## Author Summary

The architecture of vascular networks must balance complex demands to efficiently deliver oxygen and resources throughout the entire body. These demands constrain the possible forms of vasculature. Because of these constraints and the indispensable role of vasculature for much of life, scientists have sought to identify systematic patterns in the structural properties of vascular networks and whether these patterns can be predicted from models based on biological and physical principles. These studies have been limited by the lack of extensive, detailed data. Using high-quality vascular network data obtained via our software, Angicart, we identify novel, systematic patterns of asymmetry in sizes and branching angles among sibling vessels from mouse lung and human head and torso. To examine what constraints might underlie these patterns, we investigate several explanations, including various types of optimal branching as well as random branching. The optimal branchings were derived locally with respect to constraints on material costs or power loss. For random branching we allowed the degree of randomness to vary from local to global spatial scales. By comparing predictions with real data, our study suggests that a key component in determining vascular branching is material cost with some randomness at local to intermediate spatial scales.

## Introduction

The cardiovascular system is responsible for the vital processes of delivering oxygen and nutrients to cells, as well as clearing waste products, via blood flow from heart to capillaries. Accomplishing these processes requires highly complex structures because nearly all the cells throughout the body are fed by capillaries—the terminal tips of the cardiovascular system.

These linkages explain why the cardiovascular system plays a critical role in most modern allometric scaling theories that relate metabolic rate and body mass via a power law with the scaling exponent 3/4 [1–8]. Recent analyses of allometric scaling relationships using extensive data (more than 600 mammalian species and 64 plant species) yield second-order curvature in log-log space that represent deviations from this pure power law [9–13]. Attempts to account for this observed curvature, via including higher-order approximations and more accurate fluid dynamic relations, lead to curvature in the opposite direction (convex versus concave) of the empirical data [4]. This and other recent results [14] suggest the need to revisit the assumptions behind the existing models. In this paper, we show the ways in which current assumptions are insufficient to capture the patterns in empirical vascular data, and we propose new assumptions for vascular branching that could help eventually provide the foundation for a revised allometric scaling theory.

Current theories for allometric scaling, such as those proposed by West, Brown, and Enquist (WBE) [5], Banavar et al. [15, 16], Dodds [17], and Huo and Kassab [18] rely on mathematical models that encompass the architectural design of the cardiovascular system to different degrees of detail and accuracy. Within these models, the cardiovascular system is typically idealized as a hierarchical branching network that is constrained by a few core physical and biological principles. These principles lead to derivations for fractal-like, self-similar properties—having a pattern that repeats itself across large and small scales—for the overall structure of the network [19–21]. Previous models also often assume that branching is symmetric such that sibling vessels—daughter vessels branching from the same parent—are identical in terms of their radius, length, flow rate, and pressure.

Although many models presume perfect symmetry between siblings [5, 9, 18, 22], inspections of vessel casts and images reveal that some regions have highly asymmetric branching [23–26]. Such asymmetric branching patterns were empirically quantified by Zamir who showed there are differing degrees of asymmetry across levels of the coronary arteries [21, 27]. Moreover, a recent paper by Hunt et al. [28] shows that there is a high degree of asymmetry in vessel lengths within the mouse lung (micro-CT images) and human head and torso (MRI), demonstrating that symmetric branching is not an accurate representation of the cardiovascular system. Nevertheless, if the degree of asymmetry is repeated across branching junctions, this would still represent a modified version of self-similarity.

Given the evidence for asymmetric branching, we propose to investigate patterns of asymmetry in vascular branching. Because vessel radii exhibit relatively little asymmetry and are consistent with existing models [29], we focus on asymmetries in vessel lengths and branching angles. Through the identification and investigation of these new, systematic patterns, an explanation might eventually be obtained for the mismatch between theoretical predictions from scaling theory and empirical data [9]. Conversely, the consistency of the empirical scaling relationships across different species and taxa [4, 8, 9, 11, 12] suggests that shared developmental processes and evolutionary pressures powerfully constrain the degree of asymmetric branching within the vascular system, possibly corresponding to core, yet unidentified, biological and physical principles.

Developmental processes are known to play a key role in vascular branching, and these processes likely introduce stochasticity and randomness into patterns of asymmetry in lengths of sibling vessels. For example, at local to intermediate spatial scales, vessel branching is often initiated by the metabolic demands of growing tissue as signaled by expression and concentration levels of Vascular Endothelial Growth Factor (VEGF) [30]. In addition, at even more local scales, branching location is primarily affected by the shear-stress gradient between the fluid and vessel wall, related to the curvature of the branching vessel [31, 32].

In terms of evolutionary pressures, it has been proposed that the architecture of the cardiovascular system is governed by optimization principles such as minimization of the construction materials (i.e., material cost (MC)) or of power loss (i.e., power cost (PC)) across the network to provide efficient flow [33–37]. These principles lead to largely deterministic outcomes that could constrain the branching structure locally (individual branching junction), globally (whole network), or through some intermediate spatial scale. Indeed, at the global scale, vascular branching likely requires more deterministic or programmed processes due to the necessity of distributing blood to the extremities such as hands, feet, and the brain.

**Fig 1:**
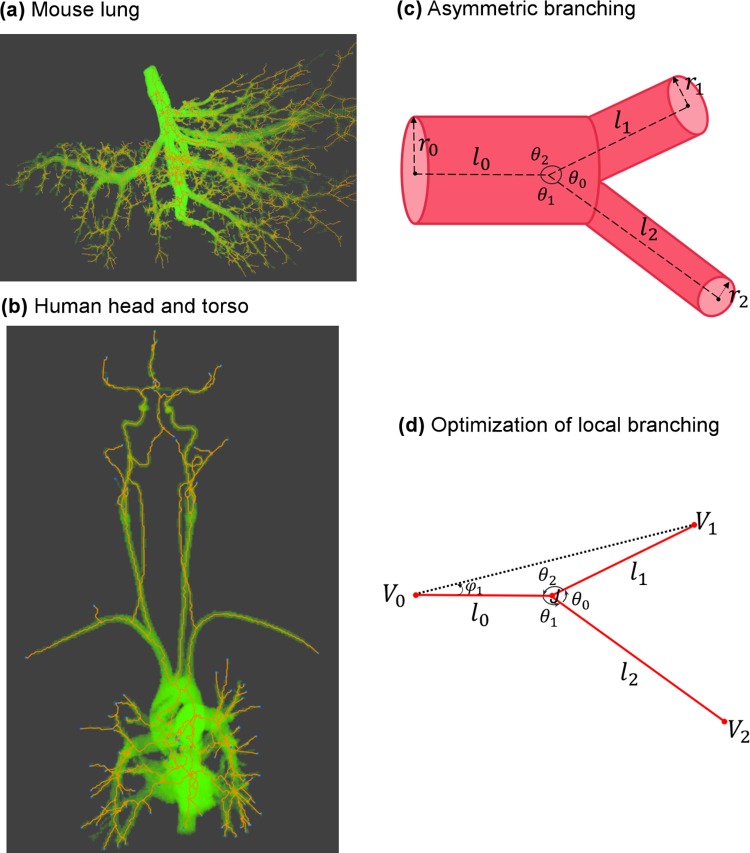
Cardiovascular data and schematic illustration of vascular branching **(a)** Mouse lung micro-CT images processed by Angicart. **(b)** Human head and torso MRI images processed by Angicart. **(c)** Schematic illustration of the asymmetric branching geometry and labeling. Parent vessel with radius *r*_0_ and length *l*_0_ branches into two daughter vessels with radius *r*_*i*_ and length *l*_*i*_ with subscript *i* = 1 or 2. Branching angles, *θ*_*i*_, are defined by the angle between the sides defined by the endpoints of the vessel pairs. Here, subscripts are determined by the non-adjacent vessel. (see Materials and Methods) **(d)** Optimization of local branching on a plane finds the optimal location of the branching junction *J* when the unshared endpoints (*V*_*i*_) and the radii (*r*_*i*_) are fixed (see General framework for branching angle optimization and asymmetry).

The local optimization of MC and PC at each branching junction—where a parent vessel branches into daughter vessels—has been studied by Murray and Zamir for the case that the radii of vessels are fixed [33, 34, 37, 38]. These optimization problems were used to derive predictions for branching angles of sibling vessels relative to the parent vessel (Fig 1C and 1D). Although not explicitly considered in previous studies, length asymmetry is directly tied to branching angles because both are completely determined by the position of the branching junction relative to the other endpoints of the vessels. Furthermore, these previous studies used a fluid-dynamically incorrect linear summation of each vessel’s individual power loss to calculate total power loss for the branching junction [33, 34]. This approach works for construction material but does not for power loss because it ignores different rules for how to combine resistances/impedances of vessels in parallel versus in series. Moreover, even for minimization of material cost, these studies [33, 34, 37] did not examine the full solution space for the optimal position of the branching junction, resulting in misleading solutions for some cases. In this paper, we address these problems and clarify the confusion in the literature on optimal branching geometries. In so doing, we provide a connection between optimal branching angles, optimal ratios and asymmetries for sibling vessel lengths, and observed asymmetry in branching patterns.

Although there are substantial theoretical predictions for the vascular system, those predictions have rarely been tested on an extensive set of data. Most vascular data have been collected via casting or dyeing methods [39–42] that do not produce sufficiently detailed data or large enough amounts of data due to the challenges of manually measuring branching angles. Recently, a novel software package, Angicart, was developed to extract three-dimensional vascular networks from the aligned stacks of high quality angiographic images [29, 43]. In this paper, we employ Angicart to analyze characteristics of the cardiovascular structure from micro-CT images of healthy mouse lung (Fig 1A) (micro-CT imaging is described in [44]) as well as the MRI of human head and torso in 18 different subjects (Fig 1B) [28, 29]. Consequently, we have collected detailed data for vascular networks that include asymmetry ratios at each junction (see Analysis of asymmetry patterns in vascular data) and measures of vessel radii, lengths, and branching angles (see Materials and Methods).

Here, we present patterns that hold across the entire network for the degree of asymmetry in vessel radius, length, and branching angle between sibling vessels. Then, we investigate the validity of the previously proposed branching angle optimizations at the local level. As part of this, we propose an alternate method for solving these minimization problems, including the full solution of the MC optimization that considers both total surface area and total volume. Next, we introduce an optimization scheme for minimizing power loss (i.e., PC optimization) that correctly implements the flow dynamics for a bifurcating structure and can also incorporate downstream impedances and power loss. We note that several of these optimization schemes result in network-level patterns of asymmetry similar to those observed in the real data. Next, as a stronger test of which optimization principles, if any, lead to the asymmetric branching patterns in real systems, we compute theoretical predictions for each branching junction in a comprehensive set of vascular data from mouse and human subjects and compare whether our predictions match the real data at the junction-level. Finally, we explore how random branching is constrained at different spatial scales, ranging from local to intermediate to global, affects the network-level characteristics of the empirical data via simulations of the branching structure. Overall, we compare our carefully constructed mathematical frameworks for optimal and random processes of vascular branching to new analyses of recent empirical data that enables us to improve our understanding of how structural properties of the vascular system are constrained by core biological and physical principles.

## Optimal Models and Random Simulations for Vascular Branching

### General framework for branching angle optimization and asymmetry

Systematic patterns in branching angles or asymmetric ratios [28, 45–47] (see Results, Analysis of asymmetry patterns in vascular data) suggest that a constraint that likely arises through natural selection, the nature of the growth process, or both. Most hypotheses about the force of natural selection on vasculature have focused on principles that reduce the cost of materials and growth while also providing efficient flow mechanisms [27, 34–37]. These biological principles could apply at each branching junction (locally) during growth or across the whole network (globally) through some larger bauplan. In this study, we initially focus on the local optimization aspects of these principles. Then, we consider applying different spatial (regional) constraints on the branching structure, including simulating networks that have random branching within constraints that range from intermediate to global spatial levels of the network.

For the local optimization of bifurcating branching geometry, following a similar approach as in the previous studies by Zamir and Murray [33, 34, 37], we assume that the radius (*r*_*i*_) and the unshared endpoints of the vessels, i.e., the vertices (*V*_*i*_), are fixed, whereas the branching junction at the shared endpoint (*J*) varies (Fig 1C and 1D). In this framework, we derive the optimal placement of the branching junction (*J*) by constructing and minimizing a cost function based on each biological principle that is hypothesized to increase the fitness. In all our derivations, we follow Krogh’s model that regards blood vessels as cylinders [48, 49]. In addition, both human and mouse data provide evidence that all of the vessels involved in a branching junction lie within a single plane (S1 Fig). Thus, for our derivations of optimal branching geometry, we assume that the branching junction lies in the plane determined by the unshared endpoints of all vessels.

To try to elucidate the cause of the high degree of length asymmetry observed in data (see Results), we also take a new approach and derive the length asymmetry from the solutions of these optimization principles. That is, by finding optimal branching angle solutions, we determine the location of the branching junction that in turn uniquely determines vessel lengths and leads to predictions for optimal length asymmetry. The formula that associates the length asymmetry with the optimal branching angles is introduced in the Appendix A.

As mentioned above, we consider two general principles. First, because construction and maintenance can be expensive to the body, minimizing total material is a potential driving factor for the structure of the vascular system. Such a principle will be referred to as *material-cost optimization* (MC optimization). The total material cost across the bifurcation is the linear sum of the material cost for each vessel because material cost is an additive quantity over different vessels.

Next, a viable design for the vascular system requires efficient flow mechanisms—such as minimal power loss—to transport nutrients and oxygen to cells. This scheme will be called *power-cost optimization* (PC optimization). In order to establish a cost function that represents the total power loss across a bifurcation, we need to use appropriate fluid-dynamical concepts. Because the effective impedance is not a linearly additive quantity for combining vessels at a branching junction, PC optimization requires a more complicated cost function than MC optimization. In this respect, our derivation differs from and corrects previous branching angle optimizations by Zamir [33, 34]. As another new element to our approach, we further propose an optimization scheme that can incorporate the power cost due to the downstream impedances beyond just a single branching junction.

In the following sections, we first introduce the branching angle optimization solutions for the MC and PC optimizations for a local branching junction. Next, we explain a new scheme for the PC optimization that incorporates information about the flow properties of the downstream vessels. Lastly, we relax the optimization principles and present simulations of random branching to explore how constraints—local to intermediate to global—can alone affect the characteristics of the vascular network.

### Material-cost (MC) optimizations

There are two types of material that are needed for the vascular system, blood vessels (endothelial cells) and blood (plasma and white and red blood cells). The amount of material necessary for vessel construction primarily depends on the surface-area of the vessel 2*πrl*. In contrast, the material devoted to the blood is proportional to the blood volume (*πr*^2^*l*). MC optimizations can be built upon these two different characteristics (surface-area (MC-SA) or volume (MC-V)). We consider both cases here.

To consider total surface-area and volume as distinct structural constraints that need to be minimized to conserve construction material, as in Murray and Zamir [33, 34, 37], we form a generic cost function *H* in which lengths are weighted according to the corresponding optimization. Explicitly, *H* = ∑_*i*_ *h*_*i*_*l*_*i*_ where *h*_*i*_ = 2*πr*_*i*_ for surface-area and *h*_*i*_ = *πr*_*i*_^2^ for volume. In general, the constant geometric factors like 2*π* can be ignored because they cancel from every term when the derivative of *H* is set equal to zero. As introduced above, we assume that the radius and the unshared endpoints for each vessel are fixed when performing the optimization calculation. For fixed radii (or equivalently for fixed cost terms *h*_*i*_) and the vertices, the MC optimization problem is equivalent to the weighted Fermat Problem introduced by Greenberg and Robertello [50–52].

To solve these minimization problems, we present a different method than that used to obtain Zamir and Murray’s solutions. Our method relies on distance metrics without invoking a coordinate system (Appendix B) and is therefore simpler and more general. The method is straightforward when one realizes that the location of the branching junction is uniquely defined by the parent vessel length (*l*_0_) and the angle (*φ*_1_) between the parent vessel and the edge determined by the unshared endpoints of itself (*V*_0_) and one of its daughter vessels (*V*_1_) (see Fig 1D). Based on this, we obtain the full solution of optimal branching angles by finding the stationary and singular points of *H* with respect to *l*_0_ and *φ*_1_ throughout the entire space.

In this framework, we recognize that the first order derivatives of *H* are discontinuous and thus undefined at the unshared endpoints (*V*_0_, *V*_1_, *V*_2_). Hence, singularities (values of infinity) are attained at these endpoints. Moreover, we find that the stationary solution for minimizing *H* exists only in the interior of the triangle defined by the unshared endpoints. In the latter case, the optimal branching angle solution is

$$\cos\theta_{0}=\frac{{h_{0}}^{2}-{h_{1}}^{2}-{h_{2}}^{2}}{2h_{1}h_{2}},\;\cos\theta_{1}=\frac{{h_{1}}^{2}-{h_{0}}^{2}-{h_{2}}^{2}}{2h_{0}h_{2}},\;\cos\theta_{2}=\frac{{h_{2}}^{2}-{h_{0}}^{2}-{h_{1}}^{2}}{2h_{0}h_{1}}$$ (1)

Note that our branching angles are defined differently than in Zamir [33, 34], so these equations are equivalent but not identical. Recognizing our definitions are relative to the parent rather than centerline extended from the parent, these expressions can be translated into Zamir’s solutions by subtracting our angles *θ*_1_ and *θ*_2_ from *π*. Moreover, in our 2D (planar) optimization scheme, we use the conventional counter-clockwise direction to define the angles so that the trigonometric functions have consistent signs (Fig 1D). Correctly defining the directionality of the angles is needed for our mathematical derivation, but for comparing to empirical data, we compute only the magnitude of the branching angles. Although it is more challenging to define directionality of angles for the real, empirical data in three dimensions, it is still straightforward to measure the magnitude of the angle between the straight lines defined by the endpoints of vessels (see Materials and Methods), allowing us to directly compare with the solutions from the 2D optimization.

Notice that the branching angle solution for the stationary case does not exist when the right side of the above expressions are not in the interval [-1,1]. For instance, when *h*_0_ = 2.1 and *h*_1_ = *h*_2_ = 1, then cos *θ*_0_ = 1.21 and *θ*_0_ is not defined. Values outside of the allowed interval occur frequently when substituting values from real vasculature, so these equations should not be blindly applied without considering the allowed regions and intervals. Moreover, there are cases for which an optimal branching solution can be computed from the above equations, but the resulting branching junction does not lie inside the triangle defined by the fixed vessel endpoints. In this case, the computed optimum does not correspond to the true optimum, which actually occurs at one of the fixed vessel endpoints (i.e., the vertices of the triangle). Indeed, for both of these cases where the stationary solution either cannot be computed or the computed answer lies outside the pre-defined triangle, the optimal solution is actually attained at one of the singularity points, i.e., the vertices *V*_*i*_.

Based on these observations, we find the conditions on the cost parameters and the geometry of the fixed endpoints that correspond to degenerate branching solutions. For example, when the cost of one vessel exceeds the total cost by the other vessels (*h*_*i*_ ≥ *h*_*j*_ + *h*_*k*_), then the optimal branching occurs at the unshared endpoint of the vessel *i* (*V*_*i*_) to eradicate this particularly costly vessel. On the other hand, when the solution *θ*_*i*_ defined by (Eq 1) is less than the angle *V*_*j*_*V*_*i*_*V*_*k*_ (i.e., cos *θ*_*i*_ > cos *V*_*j*_*V*_*i*_*V*_*k*_), which we refer to as the triangle condition, then the branching junction collapses onto the vertex *V*_*i*_. The details of the proof are given in the Appendix C.

These degeneracies are not specified in previous work [33, 34, 37] because vessels are assumed to have volume flow rates that are proportional to *r*^3^. With this presumption and by the conservation of volume flow rate across each branching junction, those studies assume a strict relationship between radii of the parent and daughter vessels (Generalized Murray’s law: *r*_0_^*d*^ = *r*_1_^*d*^ + *r*_2_^*d*^, where *d* ∈ [2,3] [36]) that avoids the degeneracy (S2 Text). Moreover, previous work does not explicitly consider cases in which the branching angle solution cannot satisfy the triangle conditions defined above. Importantly, we find that these ignored cases and conditions correspond to the vast majority of values calculated from empirical branching vessels (S2 Fig).

### Power-cost optimization for a single branching junction (PC-0)

Another biological principle and property to optimize is the power loss for pumping blood from the heart to the capillaries. This principle is tantamount to minimizing the total power for circulating blood or the total power lost that represents additional power beyond what is used to move the blood. In this way, as much of the additional power as possible is then redistributed and devoted to other needs such as foraging and reproduction [2, 5]. Much of the power that is required to push blood through the parent and daughter vessels is lost due to dissipation, especially in the smaller vessels that dominate the numbers and energetics of the whole vascular network. We calculate the power dissipated by a single vessel in terms of the volume flow rate of the blood ($\dot{Q}$) and the impedance (*Z*) via the formula $P_{loss}={\dot{Q}}^{2}Z$ [27, 35, 53]. To correctly compute the total power loss of all vessels connected by a single branching junction—a parent and daughter vessels, we must employ rules of fluid dynamics to combine impedances into an equivalent impedance. This equivalent impedance must in turn be used in the cost function for the power minimization (see below), and as we show, the correct version is different than the simple linear addition rule used by Zamir for both structural (correctly) and flow (incorrectly) constraints [33, 34].

For any collection of vessels, total volume flow rate ${\dot{Q}}_{TOT}$ is defined by flow through a single cross section that cuts through all vessels at the same branching level. Equivalent impedance *Z*_*eq*_ for the collection of vessels is defined by $Z=\Delta p/\dot{Q}$, based on fluid dynamics (analogue to Ohm’s Law), where Δ*p* is the pressure difference across the extreme endpoints through which blood flows in and out of the entire collections of vessels. The total power loss for the collected vessels is then calculated from $P_{loss}={{\dot{Q}}_{TOT}}^{2}Z_{eq}$, where all of this is based on standard rules of fluid dynamics.

For a single branching junction, the optimization of power loss is derived for the case that there is a source with a constant rate of flow, $\dot{Q}$, entering the parent vessel. Based on our power loss equation ($P_{loss}={{\dot{Q}}_{TOT}}^{2}Z_{eq}\propto Z_{eq}$), the PC optimization is then equivalent to minimizing the equivalent impedance *Z*_*eq*_ of the branching junction. By following the direction of the flow, we calculate the equivalent impedance by noting that vessels at the same level (i.e., sibling vessels) are in a parallel configuration, whereas the vessels across levels (i.e., parent and daughter vessels) are in a series configuration. Pressure drop across sibling vessels may be asymmetric but to simplify the calculation of an equivalent impedance, we further posit that the pressure drop across each daughter vessel is approximately the same, i.e., Δ*p*_1_ = Δ*p*_2_ ≔ Δ*p*_*d*_. Representing total volume flow rate of the daughter vessels by ${\dot{Q}}_{d,TOT}≔{\dot{Q}}_{1}+{\dot{Q}}_{2}$ and equivalent impedance of the daughter vessels by *Z*_*d*,*eq*_, we have Δ*p*_*d*_/*Z*_*d*,*eq*_ = Δ*p*_1_/*Z*_1_ + Δ*p*_2_/*Z*_2_. Canceling the pressure terms gives $Z_{d,eq}={\left(\frac{1}{Z_{1}}+\frac{1}{Z_{2}}\right)}^{-1}$. By invoking conservation of fluid, we can combine this expression with the parent vessel in series to obtain $Z_{eq}=Z_{0}+{\left(\frac{1}{Z_{1}}+\frac{1}{Z_{2}}\right)}^{-1}$ for the equivalent impedance of all the vessels that connect at a single branching junction.

Assuming smooth, Poiseuille flow, the impedance of a single vessel is given by $Z=\frac{8\mu l}{\pi r^{4}}$, where *μ* is the viscosity of the blood [24, 27, 53, 54]. Thus, for each vessel we implement a cost function of the form *hl*, where *h* is the cost per length, i.e., $h\equiv \frac{8\mu }{\pi r^{4}}\propto \frac{1}{r^{4}}$. Putting all of this together to find a solution for the PC-0 optimization (power loss at a single branching junction), our goal is to find the position of the branching junction that minimizes

$$Z_{eq}=h_{0}l_{0}+{\left(\frac{1}{h_{1}l_{1}}+\frac{1}{h_{2}l_{2}}\right)}^{-1}$$

By numerically calculating *Z*_*eq*_ as a function of the junction point and by using high resolution in space, we generate heat maps that illustrate the behavior of *Z*_*eq*_. These heat maps reveal that the *branching junction always collapses onto one of the vertices*—unshared vessel endpoints—for *any values* of vessel radii (S3 Fig). Based on our numerical evidence, we show analytically that power loss and equivalent impedance, *Z*_*eq*_, attain minima only at a vertex of our original triangle that is defined by the unshared endpoints of the vessel. The specific vertex at which the junction collapses is determined both by the vessel radii and the relative locations of the unshared endpoints of the vessels. The proof follows from two steps. First, we calculate the equivalent impedance at each vertex to see how specific cases of cost parameters (*h*_0_,*h*_1_,*h*_2_) determine which vertex is the best location for the branching junction *J*. Second, we show that when the junction is located within the triangle, *Z*_*eq*_ is always greater than the minimum value of the impedances when the junction is at a specific vertex. Together, this proves that the minimum of *Z*_*eq*_ is attained at one of the vertices (Appendix D). Therefore, the PC-0 optimization leads to a degenerate branching geometry by completely eliminating the vessel that is most costly.

Thus, minimizing power loss at a single branching junction leads to either no branching at all throughout the entire cardiovascular network or to a single hub at the heart with a long, individual vessel to each terminal tip—the most downstream vessel of the network. This architecture would result in extremely large numbers of vessels directly connected to the heart because terminal tips correspond to capillaries (numbering in the millions or billions) for complete networks. This is unrealistic as sequential branching is the most noticeable and perhaps most important feature of the vascular system or any resource-distribution network. Therefore, we consider how to modify the PC minimization to attain more biologically realistic results that include vascular branching and thus lead to predictions for the branching angles and vessel lengths. In the next section, we describe how to adapt the power-minimization calculation to include larger sections of the network that expand beyond a single branching junction to encompass downstream vessels and branching junctions.

### Power-cost optimization beyond a single branching junction (PC-1)

The solutions above thus lead to either a single path or to the heart being a single hub that separately connects to each capillary through a direct and independent vessel. Either configuration for vascular architecture would prevent the vascular system from efficiently distributing blood to all the downstream vessels, capillaries, and cells. To overcome these problems, we include larger sections of the vascular network by incorporating impedances of downstream vessels. To do this generically, we recognize that downstream vessels are in series with each of the daughter vessels, so we can represent the downstream impedance by adding constant terms *c*_1_ and *c*_2_ to the impedance of the daughter vessels. Thus, the equivalent impedance of the bifurcation becomes

$${\tilde{Z}}_{eq}=Z_{0}+{\left(\frac{1}{Z_{1}+c_{1}}+\frac{1}{Z_{2}+c_{2}}\right)}^{-1}$$

For the case of vessels above the capillaries, these constant terms represent the impedance from all of the downstream vessels. For the capillary case, these constant terms are still not zero, however, because they represent the minimum impedance of a capillary, which is not allowed to be zero. We now investigate different geometries for a single branching junction for which the downstream impedances *c*_1_ and *c*_2_ are constant.

We simplify this problem using two general principles. First, when the impedance of the parent vessel and the branching daughter vessels are matched, no pulsatile reflections occur and the power loss at the bifurcation is minimized [27, 53]. Second, we assume the simple case that the siblings have identical impedances and each sibling has the same number of downstream vessels. From these two assumptions, we find that the equivalent downstream impedance is much larger than single vessel impedances except for vessels close to the capillaries (Appendix E).

Because the number and small surface-areas of capillaries will again likely dominate the power loss for the network, we solve the optimization problem when the downstream impedances are large, i.e., *c*_*i*_ ≫ *Z*_*i*_. Consequently, we expand ${\tilde{Z}}_{eq}$ as a series to first order and obtain the following approximation:

$${\tilde{Z}}_{eq}∼Z_{0}+\frac{{c_{2}}^{2}}{{\left(c_{1}+c_{2}\right)}^{2}}Z_{1}+\frac{{c_{1}}^{2}}{{\left(c_{1}+c_{2}\right)}^{2}}Z_{2}+\frac{{c_{1}c}_{2}}{c_{1}{+c}_{2}}$$

We note that the constant term $\frac{{c_{1}c}_{2}}{c_{1}{+c}_{2}}$ can be ignored because its derivative is zero and hence does not influence the optimization. As a result, we want to find the location of the branching junction that minimizes

$$h_{0}l_{0}+\frac{{c_{2}}^{2}}{{\left(c_{1}+c_{2}\right)}^{2}}h_{1}l_{1}+\frac{{c_{1}}^{2}}{{\left(c_{1}+c_{2}\right)}^{2}}h_{2}l_{2}$$

The coefficients $\frac{{c_{2}}^{2}}{{\left(c_{1}+c_{2}\right)}^{2}}$ and $\frac{{c_{1}}^{2}}{{\left(c_{1}+c_{2}\right)}^{2}}$ are always less than 1, so the cost per length for the daughter vessels are diminished by these rescaling constants, thus reducing the likelihood the solution will collapse onto a daughter vessel endpoint. Defining the non-dimensionalized ratio *k* ≔ *c*_1_/*c*_2_, the optimization function becomes

$$H≔h_{0}l_{0}+\frac{1}{{\left(k+1\right)}^{2}}h_{1}l_{1}+\frac{k^{2}}{{\left(k+1\right)}^{2}}h_{2}l_{2}$$

This further implies that the cost of the daughter vessel with the larger downstream impedance is diminished less than the cost of the other daughter vessel. This pushes the optimal branching junction towards the daughter vessel with smaller downstream impedance.

The new optimization function for power loss above (i.e., PC-1) has the same form as the cost function for the material-cost (MC) optimizations, except that the costs per length for daughter vessels are rescaled by terms that depend on *k*. We therefore simplify the notation and define ${\tilde{h}}_{0}=h_{0},\;{\tilde{h}}_{1}=\frac{1}{{\left(k+1\right)}^{2}}h_{1},\;{\tilde{h}}_{2}=\frac{k^{2}}{{\left(k+1\right)}^{2}}h_{2}$. With these definitions for ${\tilde{h}}_{i}$, we can immediately use our results for Eq (1) to obtain the stationary solution for which the branching junction occurs inside the triangle of the unshared endpoints.

As for Eq 1 and the MC optimizations, the stationary solution does not exist or does not provide the minimum and the degenerate solution occurs at vertex *V*_*i*_ for the following cases: 1) ${\tilde{h}}_{i}\geq {\tilde{h}}_{j}+{\tilde{h}}_{k}$ or 2) $\cos\theta_{i}=\frac{{{\tilde{h}}_{i}}^{2}-{{\tilde{h}}_{j}}^{2}-{{\tilde{h}}_{k}}^{2}}{2{\tilde{h}}_{j}{\tilde{h}}_{k}}>\cos V_{j}V_{i}V_{k}$. These two conditions correspond to six inequalities in terms of *k*( = *c*_1_/*c*_2_) if we take all combination of *i*, *j*, and *k*. Solving these, we obtain the values of *k* that result in solutions within the triangle versus those that collapse on a vertex. Mapping these values into the *c*_1_*c*_2_-plane yields predictable lines that separate the plane into regions classified as non-degenerate (unction within the triangle defined by the vertices) or degenerate (collapse onto a vertex) solutions. Indeed, by using approximations to solve the above limiting case ($\frac{Z_{i}}{c_{i}}\ll 1$), we predict the full solution space. The validity of our approximate solutions for this PC-1 optimization problem is further explored in Results (Analytical solutions for power-cost optimization beyond single branching (PC-1)) by comparing them with numerical solutions.

### Expanding from local to global constraints for the random placement of branching junctions

The above optimization schemes are based on the local consideration of branching junctions: at each branching the unshared endpoints (*V*_*i*_) are fixed and the branching junction (*J*) is attained within the triangle of these endpoints (Fig 1D). To explore the effects of the size of the constraint region and also to observe the effects of random branching, possibly resulting from developmental processes, at different spatial (regional) constraints, we now consider relaxing the locality assumption to various degrees from fully local to fully global.

**Fig 2:**
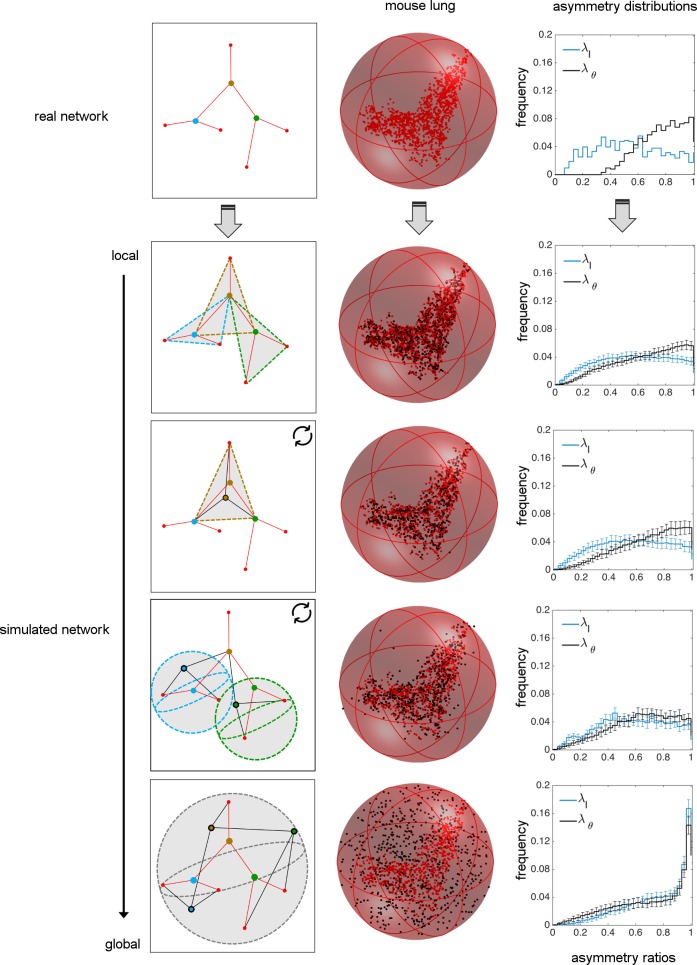
Comparison of real data for vascular networks versus random simulations of branching junctions. The real and simulated networks (via local to global spatial constraints) are separated by different rows. A schematic small network is given to describe how different simulations are performed. The vessels and the fixed endpoints of the real branching network are represented in red. Vessels that result from random branching simulations are in black. The healthy mouse lung network and the simulated mouse lung networks are shown within a minimum spherical boundary that contains all branching data from the real network. Here, the red nodes for each figure correspond to the real data, whereas the black nodes correspond to the simulated data. Note that the terminal tips and the most upstream node (i.e., the source) are determined from real data and fixed throughout all simulations. The resulting asymmetry ratio distributions for length and branching angles are provided for the real network and for each of the simulations. The statistical comparisons of random branching simulations with empirical data are given in Table 1.

We simulate the branching network by randomly placing branching junctions within spatial regions that range from local boundaries (i.e., the positions of adjacent branching junctions) to global boundaries (i.e., within some maximum distance of the center of the network but otherwise unrestricted). For all the simulations, the hierarchical ordering of vessels, the location of the terminal tips, and most upstream branching nodes (i.e., the source) are the same as in the empirical data. We created 100 realizations for each simulation type to get the average behavior of network characteristics. All these different simulation types are illustrated with a simplified example network that has 3 branching levels (Fig 2).

For the fully local case, we randomly place a branching junction inside each triangle of unshared endpoints of three connected vessels, corresponding to the same spatial restriction as in our optimizations above (Fig 2). This random branching model would likely correspond to branchings determined by locations of highest shear stress [32] or local maxima in gradients of vascular endothelial growth factor (VEGF) [30] that signal branching at local and intermediate scales. For the fully global case, we only require that the branching junctions are randomly positioned within a minimum sphere that contains all nodes from the real data [55] (Fig 2) and that the network terminates at the appropriate (most extreme upstream and downstream) endpoints in the network.

For intermediate degrees of spatial constraint between the fully local and fully global cases, we consider two possibilities. These intermediate states are constructed such that they involve sequential updates of the branching junction. The first intermediate randomly branching network simulation (intermediate 1) starts by randomly positioning a branching junction within the local triangle corresponding to the most upstream vessel (i.e., source) of the network and the endpoints of its two daughter vessels. Based on the new location of this branching junction, the endpoints of the daughter vessels are then updated and used to define new triangles in the next step in which the daughters become the parents. The simulation continues this updating process by working down through the network until it reaches the terminal tips (Fig 2, S4 Fig). Notably, this simulation leads to branching junctions that are approximately confined to the plane. The other intermediate randomly branching network (intermediate 2) starts with the terminal tips (most downstream vessels of the network) and builds backwards to the first branching node (i.e., the source). We assign the position of each branching junction by creating a spherical boundary around the two fixed downstream endpoints (i.e., *V*_1_, *V*_2_, Fig 1D) such that the center of the sphere is at the midpoint of *V*_1_*V*_2_ and the sphere has a radius of the length |*V*_1_*V*_2_|. We then randomly position the branching junction at a point that can occur anywhere within the volume of this three-dimensional sphere. Thus, for this simulation a branching junction may not always fall within the plane defined by the vessel endpoints, as it does for the first simulation for intermediately-constrained random branching. For this case, the upstream endpoint (i.e., *V*_0_) does not affect the location of the branching junction, reducing the degree of locality compared with the constraint for the simulation for intermediate 1.

## Results

We begin this section with empirical data for asymmetry in the vascular branching of mouse lung and human head and torso. Following this, we present the results of our local optimization schemes. Next, we present results for our exploration of different spatial constraints for randomly placed branching junctions and compare these results with empirical data. Finally, we provide statistical analysis of different optimization schemes and random-branching results as compared with the empirical data. This comparison enables us to quantitatively investigate how well our predictions match the empirical measurements of asymmetry in vasculature, and thus to characterize whether different optimizations or random processes might underlie the systematic patterns we observe.

### Analysis of asymmetry patterns in vascular data

To characterize the branching asymmetry of the vascular structure, we calculate asymmetry ratios between siblings. In particular, the asymmetry ratios for radius and length are *λ*_*r*_ = *r*_1_/*r*_2_ and *λ*_*l*_ = *l*_1_/*l*_2_, respectively, where the value of the sibling with the larger radius or length is always chosen to be in the denominator (*r*_1_ ≤ *r*_2_, *l*_1_ ≤ *l*_2_) [21, 27, 56]. Thus, a ratio of 1 indicates perfect symmetry, whereas smaller values indicate more asymmetrical branching. We further provide a similar measure to quantify local asymmetry in sibling branching angles as *λ*_*θ*_ = *θ*_1_/*θ*_2_ again with *θ*_1_ ≤ *θ*_2_ (Fig 1C).

It is easy to see that asymmetry in vessel lengths is related to asymmetry in branching angles. When the downstream ends of the daughter vessels are equidistant from both the upstream and downstream ends of the parent vessel, siblings have identical length and branching angles, resulting in symmetry *λ*_*l*_ = *λ*_*θ*_ = 1. However, if daughter vessels are not equidistant from the upstream end, even symmetric sibling lengths can result in asymmetric branching angles or vice versa. Therefore, the value of *λ*_*θ*_ represents a combination of the asymmetry in lengths and the asymmetry of the alignment of daughter vessel endpoints.

**Fig 3:**
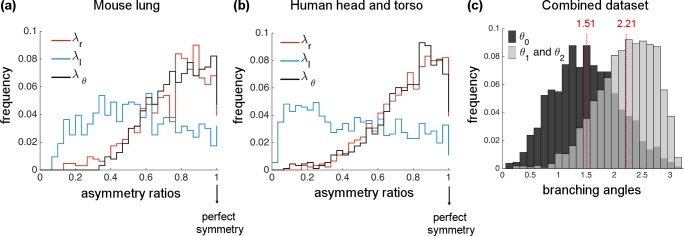
Histograms or frequency distributions of the asymmetry ratios for radius (*λ*_*r*_), length (*λ*_*l*_), and branching angles (*λ*_*θ*_) of vascular networks. **(a)** mouse lung (1 individual) and **(b)** human head and torso (18 individuals). Note that radius and branching angle asymmetry ratios are both skewed towards perfect symmetry, whereas the length asymmetry ratio shows no skew and reveals much more asymmetry. **(c)** Histograms of branching angles for combined data of human and mouse networks appear to be unimodal both for *θ*_0_ and for *θ*_1_ & *θ*_2_ with peaks at 1.51 and 2.21 radians, respectively.

After executing Angicart on high-quality tomographic images, we quantified the extent to which the analyzed networks are asymmetric by plotting the frequency distributions of *λ*_*r*_, *λ*_*l*_, and *λ*_*θ*_ (Fig 3A and 3B). Even though asymmetric radius branching exists, data for both mouse and human show distributions of *λ*_*r*_ that are skewed towards 1, meaning that the radius ratio is skewed towards perfect symmetry. In contrast, the length asymmetry ratio (*λ*_*l*_) is distributed almost uniformly, suggesting a high degree of asymmetry in sibling lengths. The frequency distribution of branching angle asymmetry (*λ*_*θ*_) is biased towards the right, corresponding to perfect symmetry in both networks and similar to results for asymmetry in radii (statistical calculations are given in Table 1). That is, a parent vessel tends to branch into sibling vessels that are separated by equal branching angles. Intriguingly, the similarity of the pattern observed in radius asymmetry might suggest a correlation between the radius and the branching angle, providing motivation to investigate how branching angle depends on radius.

**Table 1:** Statistical comparison of material-cost (MC) optimizations and random spatial constraints with empirical data.

		length asymmetry (*λ*_*l*_)				branching angle asymmetry (*λ*_*θ*_)			
		Mean± SE	SD± SE	Skewness± SE	KL p-value	Mean± SE	SD± SE	Skewness± SE	KL p-value
real network	mouse lung	0.53± 0.00	0.25± 0.00	0.13± 0.00	1.00	0.76± 0.00	0.16± 0.000	-0.43± 0.00	1.00
Material-cost (MC) optimizations	surface-area	0.54± 0.00	0.27± 0.00	-0.07± 0.00	0.01	0.82± 0.00	0.16± 0.00	-1.28± 0.00	0.00
	volume	0.54± 0.00	0.27± 0.00	-0.09± 0.00	0.00	0.74± 0.0	0.20± 0.00	-0.79± 0.00	0.01
randomly simulated networks	local	0.56± 0.01	0.25± 0.00	-0.10± 0.04	0.61	0.65± 0.01	0.24± 0.00	-0.43± 0.04	0.26
	intermediate 1	0.56± 0.01	0.25± 0.00	-0.09± 0.05	0.59	0.68± 0.01	0.22± 0.00	-0.54± 0.07	0.64
	intermediate 2	0.57± 0.01	0.24± 0.00	-0.12± 0.05	0.11	0.63± 0.01	0.23± 0.00	-0.38± 0.06	0.00
	global	0.76± 0.01	0.22± 0.01	-0.85± 0.07	0.00	0.71± 0.01	0.26± 0.01	-0.65± 0.06	0.00
real network	human head and torso	0.46± 0.00	0.28± 0.00	0.24± 0.00	1.00	0.75± 0.00	0.19± 0.00	-1.05± 0.00	1.00
Material-cost (MC) optimizations	surface-area	0.46± 0.00	0.29± 0.00	0.16± 0.00	0.76	0.85± 0.00	0.14± 0.00	-1.68± 0.00	0.00
	volume	0.48± 0.00	0.28± 0.00	0.11± 0.00	0.00	0.78± 0.00	0.19± 0.00	-0.97± 0.00	0.00
randomly simulated networks	local	0.54± 0.01	0.27± 0.00	-0.07± 0.03	0.00	0.63± 0.01	0.28± 0.00	-0.48± 0.03	0.00
	intermediate 1	0.53± 0.01	0.26± 0.00	0.00± 0.05	0.00	0.66± 0.01	0.26± 0.00	-0.64± 0.05	0.00
	intermediate 2	0.59± 0.01	0.24± 0.00	-0.24± 0.05	0.00	0.62± 0.01	0.23± 0.00	-0.34± 0.04	0.00
	global	0.75±0.01	0.22± 0.01	-0.80± 0.05	0.00	0.70± 0.01	0.26± 0.01	-0.59± 0.05	0.00

In addition to the asymmetry ratios explored above, we plot the histogram for the raw data on branching angles. Human and mouse networks show similar patterns, so we combine data for these two networks in our histogram plot (Fig 3C). Analyzing the branching angle between the sibling vessels (*θ*_0_) and the branching angle between the parent and daughter vessels (*θ*_1_ and *θ*_2_) separately, we find unimodal distributions that peak at 1.51 and 2.21 radians, respectively. This shows that planar branching [57, 58] with orthogonal daughter vessels is frequent across the networks. Individual branching angle plots for mouse and human networks are given in S5A and S5B Fig.

Altogether, these network-level patterns for vessel radius, length, and branching angles hold across 18 different human subjects, different species (human and mouse), and different tissues (head and torso versus lung). Moreover, the radius and length asymmetries are consistent with findings in plants [45–47]. These results suggest that very general and ubiquitous selection pressures and developmental processes may shape the architecture of the vascular system across taxa, from humans to mice to plants, as well as across tissues, from lungs to head and torso.

### Optimal branching patterns for material-cost (MC) optimization

In this section, we introduce the MC optimization results that include surface-area (MC-SA) and volume (MC-V) constraints. Here, we only focus on non-degenerate branching solutions—solutions that do not collapse to a vertex—and compare those with real data. The fraction of the non-degenerate and degenerate cases is provided in the S6 Fig.

### Network-level comparison

Taking the radii and vessel endpoints from our real human and mouse vascular networks, we use the material-cost (MC) optimization solution provided above (Eq 1) to compute the optimal branching angle and length asymmetry at each branching junction. To compare predicted values to the real network structures presented in the previous section, we plot the distributions of *λ*_*l*_ and *λ*_*θ*_ as well as the raw branching angle distributions for *θ*_0_ and *θ*_1_ & *θ*_2_. We find that all these properties are visually consistent across both networks and for both volume and surface-area constraints.

**Fig 4:**
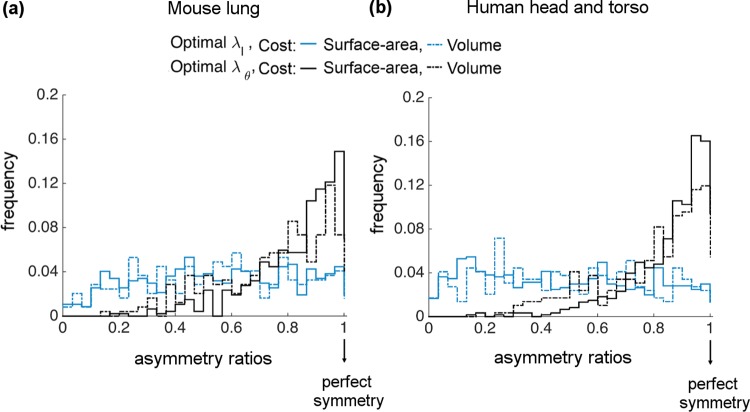
Histograms or frequency distributions of optimal asymmetry ratios for length (*λ*_*l*_) and branching angle (*λ*_*θ*_) derived from material-cost (MC) optimizations. Surface-area (MC-SA) results are shown as solid lines and volume (MC-V) results are shown as dashed lines for **(a)** mouse lung and **(b)** human head and torso.

In particular, distributions of optimal *λ*_*l*_ are close to uniform, whereas optimal *λ*_*θ*_ distributions are skewed towards perfect symmetry (Fig 4A and 4B). Both of these match the general patterns of asymmetry in branching observed in Fig 3. However, the degree of skewness in optimal *λ*_*θ*_ is sharper than the real *λ*_*θ*_ distributions, especially for the MC-SA. The statistical analysis of all these plots (including mean, median, skewness) are presented in Table 1.

**Fig 5:**
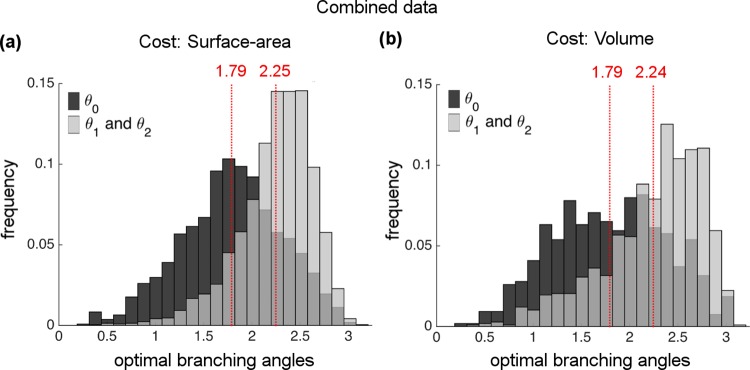
Histogram of optimal branching angles for combined data of human and mouse networks for material-cost (MC) optimizations. All histograms appear to have unimodal characteristics both for *θ*_0_ and for *θ*_1_ & *θ*_2_ with respective peaks at **(a)** 1.79 and 2.25 for the surface-area constraint and **(b)** 1.79 and 2.24 for the volume constraint.

Next, we plot optimal *θ*_0_ and *θ*_1_ & *θ*_2_ histograms for the combined datasets (Fig 5). We observe that optimal calculations yield unimodal distributions as in histograms of the real data. Optimal *θ*_0_ shows a mode around 1.79 radians, hence the optimal calculations are shifted towards larger values compared to the actual *θ*_0_ distribution that have a mode at 1.51 radians. In contrast, the peak for the uniform distribution *θ*_1_ & *θ*_2_ at 2.24 radians almost matches the actual peak at 2.21 radians. The separate figures for human and mouse networks for each constraint are given in the S5 Fig. The full statistical analysis of branching angle histograms for the individual networks and the combined datasets are provided in the S3 Table.

### Junction-level comparison

**Fig 6:**
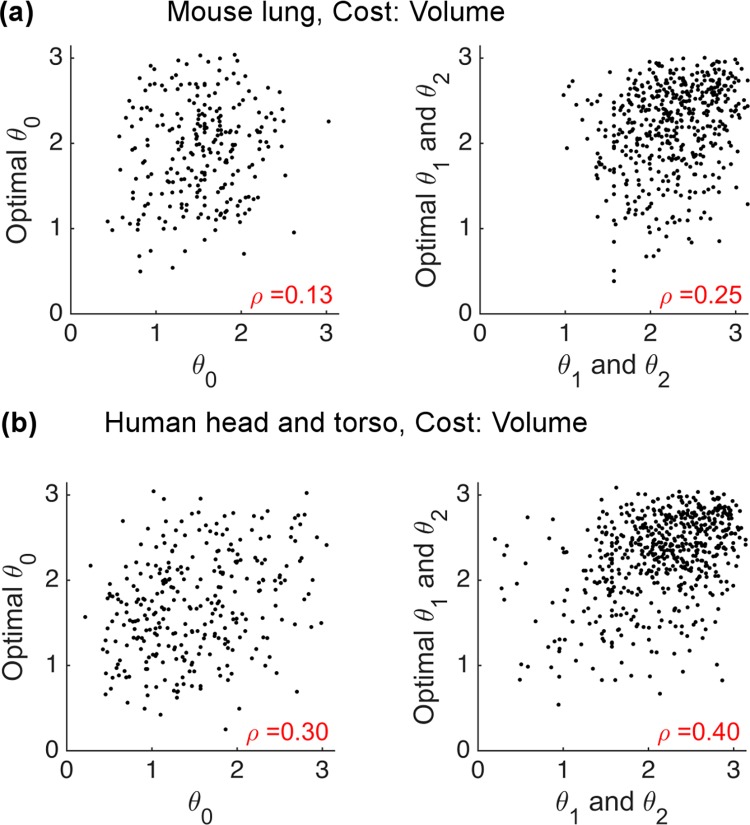
Junction-level comparison of optimal versus actual branching angles for the volume constraint of material-cost optimizations (MC-V). **(a)** mouse lung and **(b)** human head and torso. The Pearson correlation coefficients and p-values are calculated for each plot.

The previous section compares the network-level patterns of the optimal calculations and the empirical data. Here, we provide comparisons at local branching junctions for the MC optimizations. We plot actual versus optimal branching angles and calculate the Pearson correlation coefficient, which would be 1 if our predictions were always perfect. From the plots of branching angle for MC-V (Fig 6) and MC-SA (S7 Fig) optimizations, we find that the predictions and the empirical data are weakly correlated (p-values <0.01). Moreover, we see that the volume constraint yields better agreement with data than the surface-area constraint at the junction-level. However, the correlation coefficients for both constraints indicate that the predicted optimal branching angles are a weak predictor of the actual branching angles at the local junction level. Consequently, it seems our theory needs further refinement or replacement.

### Analytical solutions for power-cost optimization beyond single branching (PC-1)

Now, we introduce the results for the solution of the PC-1 optimization. As described in the section presenting the PC-1 optimization scheme, we derive the approximate solution by considering a limiting case of the downstream impedances *c*_1_ and *c*_2_. With this method our solution predicts regions in the *c*_1_*c*_2_-plane separated by lines over most of the range of values. The regions in the *c*_1_*c*_2_-plane correspond to combinations of values that lead to branching geometries that are categorized as follows: collapse to daughter endpoint, collapse to parent endpoint, or no-collapse (i.e., a non-degenerate branching point).

**Fig 7:**
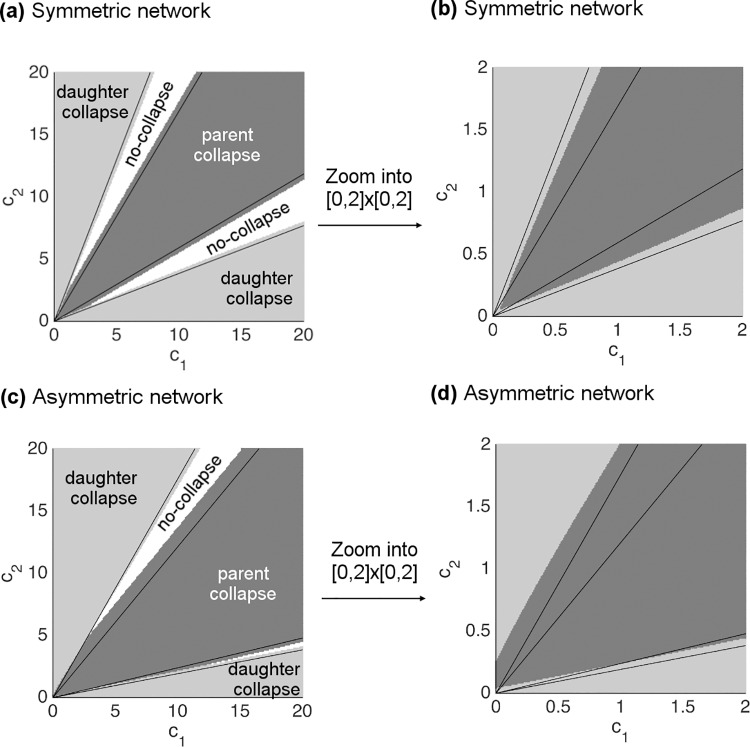
Comparison of approximate solutions with numerical solutions for the PC-1 (power-cost (PC) optimization beyond single branching). Approximate solutions define linear boundaries on the *c*_1_*c*_2_-plane between different categories of the solution space: collapse to daughter endpoint, collapse to parent endpoint, and no-collapse. The different categories calculated from numerical simulation are marked by different colors as indicated in the figure. **(a)** An example of symmetric branching in vessel radius with parameter values: |*V*_0_*V*_1_| = |*V*_0_*V*_2_| = |*V*_1_*V*_2_| = 1, r_0_ = 1.20, r_1_ = 1, and r_2_ = 1, where *c*_1_ and *c*_2_ take values in the range [0,20]. **(b)** Zoomed version of (a) into the plane [0,2] × [0,2] with the same resolution as in (a). **(c)** An example of asymmetric branching in vessel radius with parameter values: |*V*_0_*V*_1_| = 0.8, |*V*_0_*V*_2_| = |*V*_1_*V*_2_| = 1, r_0_ = 1.1, r_1_ = 0.85, and r_2_ = 1, where *c*_1_ and *c*_2_ take values in the range [0,20]. **(d)** Zoomed version of (c) into the plane [0,2] × [0,2] with the same resolution as in (c).

Using the vessel endpoint and radius information from the real data, we predict the linear equations that form the boundaries between these regions, and we label them according to the categories of solution described above (Fig 7). As a further check, we compute numerical solutions at each discretized point in the *c*_1_*c*_2_-plane and mark different categories of solution by different colors. We examine two examples, corresponding to symmetric (Fig 7A and 7B) and asymmetric (Fig 7C and 7D) parameter values. Both show that our approximate analytical solution matches the numerical solution extremely well. On closer inspection, it is clear that the different regions and categories are not separated via purely linear functions across the entire plane, but instead the boundaries are curved for small values of *c*_1_ and *c*_2_ (Fig 7B and 7D). This result reveals a mismatch between the analytical and numerical solution at the smallest scales, i.e., vessels close to the capillaries, which is exactly where our solutions should fail based on the limits of our approximation scheme.

### Network-level results of randomly branching networks with local to global constraints

Lastly, we compare our empirical results for network-level characteristics of branching angles and length asymmetries to results from simulated randomly branching networks with local to global constraints. Because mouse and human networks yield similar results, we only present results for the mouse in the main text. Random simulation results for the human subjects are given in S8 Fig.

As in the empirical data, random simulations with local to intermediate constraints yield uniform distributions for asymmetric length ratio (*λ*_*l*_), whereas the branching-angle asymmetry ratio (*λ*_*θ*_) is skewed towards symmetry (Fig 2). In contrast, the fully global constraint generates branching networks with distributions skewed towards symmetry for both *λ*_*l*_ and *λ*_*θ*_, inconsistent with empirical data (Fig 2). Further statistical comparisons with empirical data and optimal branching results are presented in the next section.

### Comparison of optimal branching, random branching, and empirical data

To provide a rigorous comparison across different MC optimizations (surface-area and volume) and spatial constraints (local to global) with the empirical data, we compute the statistical properties—mean, standard deviation (SD), skewness and standard error for each—of the resulting asymmetry distributions in Table 1. Additionally, we use the Kullback-Leibler divergence measure (KL) to quantify the distance between the empirical asymmetry distributions and the optimization or random-simulation asymmetry distributions. We determine p-values by performing bootstrap samples up to half the size of the real data [59]. By definition, a p-value is equal to 1.00 for a comparison of the real data with itself because that implies an exact match in the distributions.

There are several conclusions based on these results. First, the random branching simulations that are globally constrained do not produce results that are statistically similar with mouse lung or human head and torso in terms of length asymmetry. In addition, the intermediate 2 constraint on random branching—where spheres are used to determine the branching locations, Fig 2—poorly matches with real data in terms of both length and branching angle asymmetries for the human head and torso network.

Except for the global constraint, results for the mouse lung network reveal that the random branching simulations perform as well as the MC optimizations in terms of the general characteristics (i.e., the first few moments—mean, SD, skewness) of the distributions. In contrast, for the human head and torso, the MC optimizations yield overall better agreement with the real data than the random simulations, especially for length asymmetry. This finding is consistent with the KL significance test. Based on the KL p-values, we observe that all of the random branching simulations do not do a good job of matching the real data for human networks. However, it suggests that vascular branching derived from MC-SA does reasonably well at recreating length asymmetry patterns that are similar to real human data. In contrast, the mouse lung compares more favorably with the random-branching simulations at local and intermediate scales than it does with the MC optimizations (Table 1).

## Discussion

By performing a high-quality analysis of angiographic images from mouse lung and human head and torso via the new software Angicart [29, 43], we identified systematic patterns in the branching asymmetry of the vascular system. Specifically, the radii and branching angles of sibling vessels tend to be symmetric, while the lengths of sibling vessels tend to be asymmetric. These results show that modern allometric theories make core assumptions about symmetric branching—sibling vessels at a single branching junction have identical properties such as radius, length, and flow rate—in the vascular system that do not match empirical data, especially in terms of the asymmetry in vessel lengths. Thus, future work should be done to incorporate levels of asymmetry into theory that are similar to those observed in real data or to determine the best procedure for finding an effective, symmetrically branching network that has the same allometric properties as the asymmetric networks in real systems. Accomplishing this theoretical advance should also prove helpful in resolving the current mismatches between theoretical predictions and empirical measurements of metabolic scaling exponents. Indeed, we are involved in future work to map and compare the space of possible allometric scaling exponents both theoretically and empirically for these asymmetrically branching networks.

Importantly, the observed, systematic patterns in asymmetric vascular branching suggest there may exist underlying biological principles that vary in selection strength and across spatial scales yet effectively constrain the structure of the vascular system. In this study, we focus on MC (material-cost) and PC (power-cost) optimizations that have long been postulated as evolutionary principles that play a critical role in the formation of the vascular system [33, 34, 36, 37]. We provide a consistent and robust framework for studying these optimization principles and for discovering possible associations with the asymmetry patterns observed in the real data.

In this research, we examined local optimization and for the first time presented solutions for the MC optimization that are more complete than formulae from previous studies that are not optimal in every case or can be easily misidentified or misinterpreted. We have further shown that solutions predicted from MC optimizations match the network-level asymmetry patterns for lengths and branching angles observed in the real data but are a weak predictor of vascular branching at the junction-level. Following this, we built a PC optimization scheme that is consistent with the basic rules of fluid dynamics and corrects inconsistencies about fluid mechanics—incorrectly summing impedances in series and parallel—from previous work. Based on the correct fluid mechanics relationship, we find for single branching junctions that one vessel is always sufficiently costly to be completely eliminated and have no branching at all. Of course, repeating this solution at each junction throughout the network will result in a single vessel or a single hub at the heart for the entire cardiovascular network, which is unrealistic because it violates the need to efficiently distribute blood throughout the body [15, 16, 27, 35, 36]. Consequently, based on our new analysis, we conclude that optimization of flow and power loss at a single branching junction (i.e., PC-0 optimization) will always lead to the elimination of branching and thus lead to meaningless predictions of angles and lengths of vessels in branching networks.

We also note that another problem with local optimization is that it is not robust for these more complicated branching architectures. That is, the optimization of the power cost through a whole network (global) would be affected by changes in the flow dynamics at even a single local branching [60]. Hence, obtaining an efficient distribution of flow to optimize energy cost across a whole network would necessitate global changes in network structure if there is even a single change at the local level of a branching junction. This effect is considered by Katifori et al. [61] when deriving optimal leaf venation networks that provide robustness to damage as an evolutionary advantage for leaf survival.

In an attempt to connect vascular branching asymmetry with the efficient flow mechanisms, we included multiple downstream vessels and branching junctions as part of the PC optimization (i.e., PC-1 optimization) to determine the optimal placement of each individual branching junction. This in turn indicates the need to consider constraints beyond just the local spatial and branching junction scale. Although this is additional work, it has the advantage that capillaries are at a uniform pressure so that the full downstream vascular tree below each daughter vessel has a symmetric pressure drop, thus simplifying the optimization problem. Alternatively, the model could be improved by allowing asymmetric pressure drops across sibling vessels that could lead to plausible (i.e., non-degenerate) solutions even when locally optimizing the flow. However, determining these pressure drops would also require considering effects from multiple branching junctions when calculating the placement of a single branching junction. Furthermore, our results from these more intermediate-scale calculations reveal that plausible explanations and predictions for branching angles and length asymmetry can be obtained by incorporating vessels beyond a single branching junction. Thus, from any perspective, we argue that local-scale optimization principles and constraints are insufficient to understand and predict asymmetric branching patterns, which is in strong contrast to many previous results for symmetric branching that can be fully treated at the local scale or at a single branching junction.

Although the MC optimization results match empirical data at the network-level, the junction-level comparisons show that a strictly local constraint is unlikely to be the major driving determinant of the vascular structure. Further evidence for the possible inadequacy of local constraints is the fact that the PC optimization scheme does not lead to any realistic branching at all. Therefore, we enlarged the scale of spatial constraint and the number of branching junctions involved in the optimization in order to incorporate more information.

There is no a priori reason that evolutionary constraints should only apply at local spatial scales, and as just explained, our optimization results suggest that local spatial constraints do not fully explain the existence of the observed asymmetric branching patterns. Moreover, developmental processes likely occur at local to intermediate scales but in a more random manner than evolutionarily programmed branching in response to optimality constraints. Thus, to investigate the role of randomness and of spatial constraints on vascular structure, we considered random simulations of the branching network with spatial constraints that varied from local to global, including two types of constraints at intermediate scales. We found that the global constraint performs poorly, whereas the intermediate or local constraints provided reasonable matches to the network-level observations from real data. Here, the intermediate constraints are especially important to consider because they incorporate the downstream impedance as in our improved PC optimization scheme.

Our statistical analysis compares network-level asymmetry from empirical data to optimal predictions and randomized results. We show that random simulations and MC optimizations capture important features of the vascular branching for the mouse lung network. For the human head and torso, we find that only MC optimizations succeed at capturing the dominant features of asymmetry in vascular branching. This difference as in result from the mouse lung vasculature may arise because the human head and torso data were obtained using MRI and correspond to vessels of larger sizes that must deliver blood from the heart to the rest of the body and thus may be more programmed to follow a defined branching pattern. In contrast, the smaller-sized vessels imaged by micro-CT in the mouse lung may be filling space but with a structure that requires weaker constraints on the patterns of asymmetry in vascular branching. These smaller vessels are also likely formed due to developmental processes with more random spatial constraints such as branching triggered by VEGF signaling or locations determined by the point of the highest gradient in shear stress. Lastly, our results are intriguing because an intermediate spatial scale has been found to be the correct spatial scale for recreating pervasive patterns founds in urban studies on cities [62].

Finally, we note other attempts for understanding the architecture of the vascular systems or other types of transportation networks. In modern allometric theories with symmetric vascular branching, space-filling principles [4, 5]—a simple encapsulation of the need of the vascular system to span the entire body and have capillaries close to all cells—is the core assumption that determines the distribution and scaling of vessel lengths. Algorithms [28] have been proposed that are space filling and reproduce some of the asymmetric patterns described here. These algorithms optimize at both local and global scales within non-spherical spaces. That research does not explicitly include radius information so joining these approaches into a single framework may be a fruitful future direction. Another study by Durand et al. [63] is also noteworthy for its analysis of optimal transport networks that minimize the resistance to flow in a special case of a constant total volume and surface area across the network.

Taken together, our findings suggest that combinations of biological principles that are applied at the intermediate and local level could eventually lead to the systematic patterns for branching angles and length asymmetry observed in real data. We infer that physical constraints, developmental processes, evolution and optimization principles play a role in determining vascular structure, but in contrast to previous work [5, 15, 16, 18, 34, 37], we find that the strength and relative importance of these roles likely also depends on the spatial scale, number of branching junctions, vessel sizes, and possibly tissue type. As a result, our work suggests further exploration of optimal branching at local and intermediate spatial scales in a way that combines and integrates multiple optimization principles.

## Materials and Methods

### Processing of angiographic images, vessel extraction with Angicart software, and resolution of data

In our study we analyze the cardiovascular structure of mouse lung and human head and torso by processing the three-dimensional stacks of images via the software Angicart [29]. Mouse lung images are collected through the microtomography (micro-CT) [44], whereas human head and torso images from 18 different subjects are obtained through MRI [29, 43]. The detailed image acquisition for each dataset are given in [44] and [29]. Here, we present how these images are processed to extract the vascular geometry. Before processing the tomographic images in Angicart, the images are first downsampled to reduce the noise and decrease the processing time. Angicart relies on the manual input of the intensity threshold parameter between 0 and 1 to identify the set of voxels belonging to the vascular network. For each set of data, the intensity threshold has been chosen by the visual inspection of blood vessels, conducting the sensitivity analysis as well as identifying the percentage of the possibly misclassified vessels as presented in Newberry et al. [29]. All these steps are done to smooth or reduce the noise in the data. The details on the preprocessing steps, set of thresholds, as well as the version of the software in regards to these criterions are given in the Supplementary Material. Performing Angicart on the angiographic images extracts the branching network topology with the vessel characteristics such as positional coordinates, radius, length, number of children. The output of the Angicart for each dataset is available in S1 Dataset.

We also note that different imaging modalities can lead to different imaging qualities and that the level of spatial scales that they can identify varies. Because micro-CT provides higher resolution images than MRI, the vessel sizes identified in the mouse lung are substantially smaller than the vessel sizes identified in the human head and torso data (>10 microns versus >1mm). These differences have the advantage of allowing us to investigate the branching geometries for large versus small vessels as well as pulsatile versus viscous dissipation flow regimes [4, 27, 35]. Moreover, the difference in the quality of different medical imaging techniques inevitably affects the amount of noise in the data. Possibly misidentified vessels from the Angicart output are characterized by defining non-deformed vessels—more than 10% of the volume of voxels of the vessel lies inside a distance of radius+1 voxel from the centerline of the corresponding vessel. We find that the fraction of carefully classified, non-deformed vessels is greater in the mouse lung data compared to the human head and torso data (S2 Table), meaning it is of higher quality on average, as expected from the image resolution properties of micro-CT and MRI. This realization makes it especially noteworthy that the mouse lung data exhibits the strongest signal of random branching, which we can confidently say is not due to effects of noise but to actual branching patterns (see Results, Comparison of optimal branching, random branching, and empirical data).

### Computing branching angles from extracted vessel skeleton

To calculate our three branching angles (Fig 1C) using our empirical vessel measurements, we first must specify the three intersecting lines that define the angles. One endpoint is common to all three lines, representing the branching junction (*J*) that connects the parent to the two daughter vessels. Therefore, the remaining choice is in how to define the other endpoint of all three vessels. In principle, this could be done using points along the vessel that are very close to the branching junction (*J*), the midpoint between the branching junction and the other vessel endpoint, or the exact endpoint of the other vessel. For a perfectly straight vessel, this choice will have no effect on the computed branching angle, but for curved or tortuous vessels, which are common in real vascular systems, these choices will lead to different values for the branching angles. For this study, we choose the exact endpoints of the vessels to define the straight lines that define the angles (S9 Fig). We do this for three reasons. First, we relate branching angles to vessel lengths, which are defined relative to the endpoints, so these relations will be most faithful if we use lines corresponding to the full length of the vessel. Second, we argue there is more of a constraint on the endpoints of vessels than the exact path they take to reach those points, which may include more developmental stochasticity. Third, there is no arbitrariness to the choice of vessel endpoints, whereas choosing 1 pixel versus 5 pixels away from the branching junction is more subjective. Using our choice of lines and endpoints, we calculate the magnitude of the angles between the straight lines defined by the positional coordinates—the endpoint coordinates of the vessels *V*_0_, *V*_1_, *V*_2_, and *J*—at the bifurcation as shown in Fig 1C and 1D.

## Supporting Information

S1 Appendix Detailed proofs for the branching angle optimization solutions. (PDF)

S1 Fig Planarity of local branching junctions in mouse and human networks. When the parent and child vessels are colocalized in the same plane, the junction is considered planar. **(a)** We quantify the planarity measure of local branching is by the scaled distance formula given here. When the scaled distance is equal to 0, the branching is perfectly planar. Larger values of the scaled distance formula imply stronger deviations from planarity. **(b-c)** Histograms of the scaled distance for (b) the mouse lung and (c) the human head and torso are skewed towards the perfectly planar case, providing evidence that the local branching is mostly planar for both networks.
(PDF)

S2 Fig Type of optimal branching solution and analysis of Murray’s Law. Non-degenerate solutions (branching occurs) are specified with white, and degenerate solutions (junction collapses to vessel endpoint so no branching) are specified with black. The red color represents the region where the Generalized Murray’s law holds (scaling exponent *d* ∈ [2,3]), which is a subset of the region of non-degenerate region. **(a)** Material cost: Surface-area, **(b)** Material cost: Volume, **(c)** Analysis of vasculature for mouse lung shows that the Generalized Murray’s Law hold only for 10% (= 66/633) of the branching data, **(d)** Analysis of human head and torso shows that the Generalized Murray’s Law holds only for 13% (= 122/914) of branching data.
(PDF)

S3 Fig Heat map (color map) representation of the equivalent impedance (i.e., *Z*_*eq*_) for PC-0 (power-cost optimization for a single branching junction). The optimal branching location, J, for different choice of cost parameters (*h*_*i*_) and end points (*V*_*i*_) is marked with a green dot. The optimal branching junction coincides with the end point *V*_0_ in panel (a), *V*_1_ in panel (b), and *V*_2_ in panel (c). This highlights the fact that, depending on both the vessel cost and the geometry of the endpoints, the branching junction can collapse on the parent or either of the daughter vessel endpoints.
(PDF)

S4 Fig Iterations for randomly branching networks with intermediate constraints. The vessels and the fixed end points for the real branching network are in red. The random branching simulations start from this real networks, and newly resulting branching locations and vessels are shown in black. (1) The first simulation starts by randomly positioning the most upstream branching junction of the network (green) within the corresponding triangle of end points (green dashed triangle). The simulation iteratively continues this process by updating the new branching junction and determining the branching junctions at the next level (blue and purple) in the same way. Note that this first intermediate constraint places the branching point onto the plane of the current vessel end points, hence constraining all three vessels at a branching junction to be confined to a single plane. (2) The second simulation starts by determining the locations of the most downstream branching junctions (blue and purple) of the network. The first iteration is to randomly position new branching junctions within a spherical boundary (blue and purple dashed spheres) whose size is proportional to the distance between the two downstream endpoints (i.e., terminal tips) of the current branching junction. The simulation then iteratively builds backwards by randomly placing branching junctions within corresponding spherical boundaries at each branching generation until the first branching node is reached. These spherical boundaries relax the assumption of local planarity and lead to branching at the local level for which the three vessels connected at a branching junction do not need to be co-planar.
(PDF)

S5 Fig Histogram of branching angles and optimal branching angles of MC (material-cost) optimizations for mouse and human networks. All histograms show a unimodal distribution with means given by the red vertical lines.
(PDF)

S6 Fig Fraction of degenerate and non-degenerate branching solutions for material-cost optimizations. The surface-area constraint leads to degenerate solutions for **(a)** 26% (= 163/633) of branching junctions in the mouse lung network and **(b)** 34% (= 309/914) of branching junctions for the human head and torso network. Volume constraint leads to degenerate solutions for **(c)** 61% (= 388/633) of branching junctions in the mouse lung network and **(d)** 68% (= 621/914) of branching junctions for the human head and torso network.
(PDF)

S7 Fig Junction-level comparison of optimal versus actual branching angles for the surface-area constraint of material-cost optimizations. Results for **(a)** mouse lung and **(b)** human head and torso. The Pearson correlation coefficients are calculated for each plot.
(PDF)

S8 Fig Comparison of real data for vascular networks versus random simulations of branching junctions for human head and torso network. The real and simulated networks (via local to global constraints) are separated by different columns. A schematic small network is given to describe how different simulations are performed. The vessels and the fixed end points of the real branching network are represented in red. Vessels that result from random branching simulations are in black. The real human head and torso network and the simulated human head and torso networks are shown within a minimum spherical boundary that contains all branching data from the real network. Here, the red nodes for each figure correspond to the real data, whereas the black nodes correspond to the simulated data. Note that the terminal tips and the most upstream node (i.e., the source) are determined from real data and fixed throughout all simulations. The resulting asymmetry ratio distributions for length and branching angles are provided for the real network and for each of the simulations. The statistical comparisons of random branching simulations with empirical data are given in Table 1.
(PDF)

S9 Fig Computing branching angles from extracted vessel skeleton. We calculate branching angles as angles between the straight lines defined by the positional coordinates of the vessels—the endpoint coordinates *V*_0_, *V*_1_, *V*_2_, and *J*—at the bifurcation.
(PDF)

S1 Table The list of intensity thresholds and the version of Angicart utilized in this study and Newberry et al. [29]. (PDF)

S2 Table The percentage of non-deformed vessels in the Angicart outputs of human head and torso and mouse lung data. A vessel is identified as non-deformed when the number of voxels outside a distance rad+1 from the centerline of the vessel is less than the 10% of the total voxels of the vessel segment [29, 43].
(PDF)

S3 Table Statistical analysis of branching angle and optimal branching angle distributions from material-cost (MC) optimizations for mouse, human, and combined networks. (PDF)

S1 Text Details on the empirical data and the choice of parameters. (PDF)

S2 Text Murray’s law and optimal branching angle solutions. (PDF)

S1 Data Supplementary Data. (XLS)
