# Supplementary material for: Do Vascular Networks Branch Optimally or Randomly across Spatial Scales?
Source: PLoS Comput Biol. 2016 Nov 30;12(11):e1005223. doi: 10.1371/journal.pcbi.1005223 (PMC5130167; doi:10.1371/journal.pcbi.1005223)
Supplement: S1 Appendix — (PDF) [file pcbi.1005223.s001.pdf]

Appendix A. Derivation of Length Asymmetry Ratio (λ_l) Given Branching Angles,

Here, we relate the length asymmetry ratio (λ_l) to the optimal branching solutions (θ_i) and the geometry of the unshared endpoints (i.e., the vertices V_i). We denote the lengths of the sides of the triangle that correspond to V_1V_2, V_0V_2, and V_0V_1 as $v_0, v_1,$ and v_2, respectively (Fig A1). We first prove two Lemmas that lead to the derivation of the asymmetric length ratio.

Lemma 1: Let the intersection of the line between the points V_0 and J with the line V_1V_2 be called K and the angle defined by the three points $\widehat{V_0KV_1}$ be called ψ (Fig A1). Using these definitions and the other labeling in Fig. A1, the following relationships holds

$$\frac{|V_1K|}{|V_2K|} = \frac{l_1 \sin\theta_2}{l_2 \sin\theta_1} = \frac{v_2 \sin\varphi_1}{v_1 \sin\varphi_2}$$

Proof: By the law of sines applied to the triangles V_0V_1K and V_0V_2K, we have:

$$\frac{\sin\psi}{v_2} = \frac{\sin\varphi_1}{|V_1K|}, \qquad \frac{\sin(\pi-\psi)}{v_1} = \frac{\sin\varphi_2}{|V_2K|}$$

Since $\sin(\pi - \psi) = \sin\psi$, dividing these equations yields $\frac{|V_1K|}{|V_2K|} = \frac{v_2 \sin\varphi_1}{v_1 \sin\varphi_2}$. Applying a similar approach to triangles JV_1K and JV_2K, we have $\frac{|V_1K|}{|V_2K|} = \frac{l_1 \sin\theta_2}{l_2 \sin\theta_1}$, as desired. ∎

Figure A1. (a) Schematic of the branching geometry **(b)** Illustration of degenerate cases where the branching junction coincides with one of the vertices.

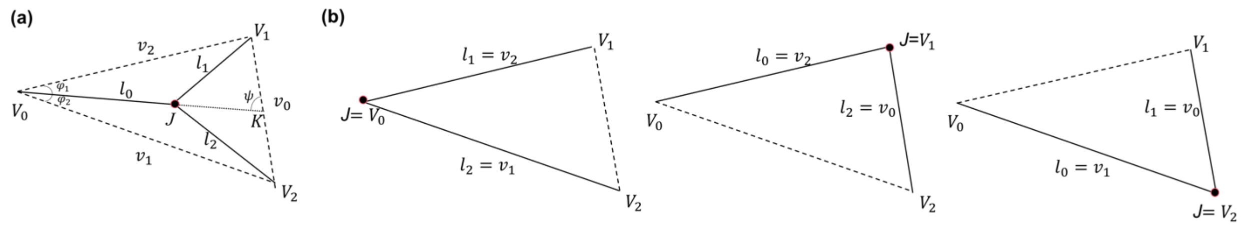

Lemma 2: The length asymmetry ratio ($\lambda_l = \frac{l_1}{l_2}$) can be calculated purely in terms of the lengths of the sides of v_1 and v_2 along with the angle $\widehat{V_1V_0V_2}$ and the branching angles θ_1 and θ_2 as

$$\lambda_l = \frac{v_2}{v_1}\frac{\sin\theta_1}{\sin\theta_2}\left(-\cos\widehat{V_1V_0V_2} + \sin\widehat{V_1V_0V_2}\cot\left(\widehat{V_1V_0V_2} + \gamma - \theta_2\right)\right)$$

where $\gamma = \cot^{-1}\left[\frac{\frac{v_2\sin\theta_1}{v_1\sin\theta_2} + \cos(\theta_1+\theta_2-\widehat{V_1V_0V_2})}{\sin(\theta_1+\theta_2-\widehat{V_1V_0V_2})}\right]$

Proof: By Lemma 1, we have

$$\lambda_l = \frac{l_1}{l_2} = \frac{v_2}{v_1}\frac{\sin\theta_1}{\sin\theta_2}\frac{\sin\varphi_1}{\sin\varphi_2}$$

Then, by applying law of sines in a specific, successive order and also using sine addition formulas, we express $\frac{\sin\varphi_1}{\sin\varphi_2}$ in terms of known quantities and branching angles:

$$\frac{\sin\varphi_1}{\sin\varphi_2} = \left(-\cos\widehat{V_1V_0V_2} + \sin\widehat{V_1V_0V_2}\cot\left(\widehat{V_1V_0V_2} + \gamma - \theta_2\right)\right)$$

proving the lemma. ■

With Lemma 2, we show that the branching angle solution—obtained by optimizing certain structural principles—also predicts the optimal value for the asymmetric length ratio.

Appendix B. Coordinate-Free Framework for Material Cost Optimization Solutions

In this section, we introduce a coordinate-free framework for the minimization of the objective function, defined as $H = \sum_i h_i l_i$. We have not seen this approach in the literature, and other references have used methods that rely on specific choices of coordinate systems and complicated algebra (1-3). The solution is obtained via finding the stationary and singular points of the cost function H with respect to l_0 (the parent vessel length) and φ_1 (the angle of the parent vessel relative to its unshared endpoint V_0) (Fig A1). Below, we provide two lemmas that will be used to determine $\frac{\partial H}{\partial l_0}$ and $\frac{\partial H}{\partial \varphi_1}$.

Lemma 3. Given fixed endpoints V_0, V_1, and V_2, the length $|V_0V_1|$ and the angle φ_1 are fixed in the triangle V_0JV_1, (Fig A2), the derivative of a daughter vessel length with respect to the parent vessel length is

$$\frac{\partial l_1}{\partial l_0} = \cos\theta_2$$

Proof: Draw a perpendicular line passing through V_1 and intersecting with the extension of V_0J at O. Denote $|V_0V_1| = v_2$, $|V_1O| = y$, and $|JO| = x$. When J is on the right side of V_0, we have $v_2 \cos\varphi = x + l_0$. Since $v_2 \cos\varphi_1$ is fixed because v_2 and φ_1 are fixed, it follows that $\partial(v_2 \cos\varphi) = \partial(x + l_0) = 0$, or equivalently

$$\frac{\partial x}{\partial l_0} = -1. \qquad \text{(A1)}$$

Notice however that the derivative $\frac{\partial x}{\partial l_0}$ is discontinuous when the branching junction collapses on the parent endpoint (i.e., $l_0 = 0$) as the right and left derivatives of x with respect to l_0 are opposite in sign: $\partial_+ x(0) = \frac{\partial(v_2 \cos\varphi_1 - l_0)}{\partial l_0} = -1,\ \partial_- x(0) = \frac{\partial(v_2 \cos\varphi_1 + l_0)}{\partial l_0} = 1$ (Fig A2).

Figure A2. (a) The branching geometry of a parent and one of the daughter vessels **(b)** When the vertex J approaches the vertex V_0 from the right, $x = v_2\, cos\, \varphi_1 - l_0$. **(c)** When the vertex J approaches the vertex V_0 from the right, $x = v_2\, cos\, \varphi_1 + l_0$.

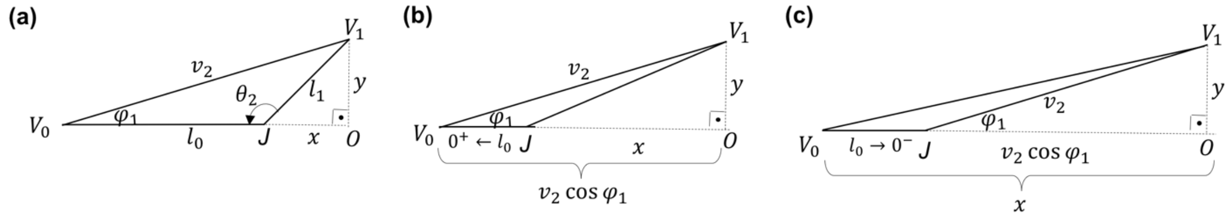

Applying the Pythagorean Theorem to the triangle V_1JO, we have $l_1 = \sqrt{x^2 + y^2}$, hence

$$\frac{\partial l_1}{\partial x} = \frac{x}{\sqrt{x^2 + y^2}} = \frac{x}{l_1} \qquad \text{(A2)}$$

Using the chain rule along with equations (A1) and (A2) gives

$$\frac{\partial l_1}{\partial l_0} = \frac{\partial l_1}{\partial x}\frac{\partial x}{\partial l_0} = -\frac{\partial l_1}{\partial x} = -\frac{x}{l_1} = -\cos(\pi - \theta_2) = \cos\theta_2$$

as desired.

Lemma 4. Given fixed lengths $|V_0V_1| = v_2$ and l_0 in the triangle V_0JV_1, then

$$\frac{\partial l_1}{\partial \varphi_1} = -l_0 \sin \theta_2$$

Proof: As in Lemma 1, we have $\cos \theta_2 = -\cos(\pi - \theta_2) = -\frac{x}{l_1}$ and $l_1 = \sqrt{x^2 + y^2}$. From the triangle V_0V_1O, we further have $y = v_2 \sin \varphi_1$ and $x = v_2 \cos \varphi_1 - l_0$. Substituting these into the expression for l_1 yields $l_1 = \sqrt{(v_2 \cos \varphi_1 - l_0)^2 + (v_2 \sin \varphi_1)^2}$. As v_2 and l_0 are fixed, differentiating l_1 with respect to φ_1 gives:

$$\frac{\partial l_1}{\partial \varphi_1} = \frac{1}{2} \frac{2(v_2 \cos \varphi_1 - l_0)(-v_2 \sin \varphi_1) + 2v_2^2 \sin \varphi_1 \cos \varphi_1}{\sqrt{(v_2 \cos \varphi_1 - l_0)^2 + (v_2 \sin \varphi_1)^2}}$$

This expression simplifies by cancelling the $2v_2^2 \sin \varphi_1 \cos \varphi_1$ terms in the numerator and by recognizing the denominator is equal to l_1. Therefore, we obtain $\frac{\partial l_1}{\partial \varphi_1} = \frac{l_0 v_2}{l_1} \sin \varphi_1$. Since $\sin \varphi_1 = \frac{y}{v_2}$ and $\sin(\pi - \theta_2) = \frac{y}{l_1}$, this equation becomes

$$\frac{\partial l_1}{\partial \varphi_1} = \frac{l_0 v_2}{l_1} \sin \varphi = l_0 \frac{y}{l_1} = -l_0 \sin \theta_2 \ \blacksquare$$

With these two lemmas proven, we now return to the original optimization problem. Unless *J* coincides with the unshared endpoints V_0, V_1 or V_2, substituting Lemma 1 and Lemma 2 into the equality, we have

$$\nabla H = \left(\frac{\partial H}{\partial l_0}, \frac{\partial H}{\partial \varphi_1}\right) = \vec{0},$$

leads to two equations

$$h_0 = -h_1 \cos \theta_2 - h_2 \cos \theta_1 \qquad \text{(A3)}$$

$$h_1 \, sin\, \theta_2 = h_2 \, sin\, \theta_1 \qquad \text{(A4)}$$

Solving these equations yields the previously reported branching angle solutions (Eq. (1) in our paper and from Zamir et. al. (1, 2)).

Dividing both sides of the equations (A3) and (A4) by h_2 and combining them, we have

$$\frac{h_0}{h_2} = -\frac{\sin\theta_1 cos\theta_2 + \sin\theta_2 cos\theta_1}{\sin\theta_2} = \frac{-\sin(\theta_1+\theta_2)}{\sin\theta_2}$$

Realizing that $\theta_1 + \theta_2 = 2\pi - \theta_0$, or equivalently $-\sin(\theta_1+\theta_2) = \sin\theta_0$, and combining the above equations (A3) and (A4) yields $h_0 \, sin\, \theta_2 = h_2 \, sin\, \theta_0$. Thus, in order for the equations that follow from $\nabla H = \vec{0}$ to be soluble, the expressions $\sin\theta_0$, $sin\,\theta_1$, and $\sin\theta_2$ must all have the same sign because the length scales h_i are all positive. This sign criterion can only be satisfied when the branching junction is inside of the triangle defined by V_0, V_1 and V_2. Consequently, this implies $\nabla H = \vec{0}$ cannot be satisfied when the branching junction is outside of the triangle or on the boundary of the triangle. Therefore, in order for the previously reported formula for the branching-angle solutions to be valid, we need to check first if $-1 \leq \cos\theta_i \leq 1$, and if it does not, we must conclude that $\nabla H = \vec{0}$ does not have a solution. Previous studies were not explicit about this criterion or distinction in finding solutions. Solving the inequalities $-1 \leq \cos\theta_i \leq 1$ for each branching angle yields necessary conditions for the existence of solutions of $\nabla H = \vec{0}$. These conditions reduce to the simple statement, $h_i < h_j + h_k$, about the weightings of the terms in the cost function for any combination of (i, j, k). If any of these

three conditions fail, then the branching junction will be degenerate, meaning that the optimal branching junction, J, will collapse to one of the vertices.

Moreover, the angles of the triangle $V_0V_1V_2$ further confine the range of branching angles that can be realized within the triangle, i.e. $\widehat{V_jV_iV_k} < \theta_i$. Hence, if branching angle solutions defined by Eq. (1) violate any of these conditions, the optimization solution will be a collapse of the branching junction onto one of the unshared endpoints.

Appendix C. Degeneracy Solutions of Material Cost Optimization

We now derive which particular vertex the branching junction will collapse onto for the degeneracy cases.

Lemma 5. When the triangle conditions and inequalities do not hold (i.e., $h_i \geq h_j + h_k$), the vertex V_i associated with the largest cost parameter (i.e., h_i) is the solution for the material cost optimization.

Proof: By symmetry and without loss of generality, we assume that the cost per parent length is greater than the sum of the costs per length for the daughter vessels, i.e. $h_0 \geq h_1 + h_2$. To identify the vertex that yields the minimum cost, we will calculate the total cost corresponding to all three degenerate cases (Fig A1). Total costs at the corresponding vertices are given by $H_{V_0} = h_1v_2 + h_2v_1, H_{V_1} = h_0v_2 + h_2v_0, \text{and } H_{V_2} = h_0v_1 + h_1v_0$, where $v_0, v_1,$ and v_2 are lengths of sides V_1V_2, V_0V_2, V_0V_1 respectively. From our assumption and triangle inequality applied to the sides of the triangle $V_0V_1V_2$, we have $H_{V_1} = h_0v_2 + h_2v_0 \geq (h_1 + h_2)v_2 + h_2v_0 = h_2(v_0 + v_2) + h_1v_2 > h_2v_1 + h_1v_2 = H_{V_0}$. In a symmetric way, one can also prove that $H_{V_2} > H_{V_1}$, implying that J collapses on V_0.

Lemma 6. For any triangle with vertices X, Y, Z, and a point P inside this triangle we have the following inequality

$$|XY| + |YZ| > |XP| + |PZ|$$

Proof: The set of points Y’ on the plane for which

$$|XY'| + |Y'Z| = |XY| + |YZ|$$

forms an ellipse as illustrated in Fig A3. Therefore, for any point P' in the interior of the ellipse

$$|XP'| + |P'Z| < |XY| + |YZ|$$

proving the claim.

Fig A3. Ellipse formed by the points X, Y, and Z. By definition, the sum of the distances from any point on the ellipse to X and Z is fixed.

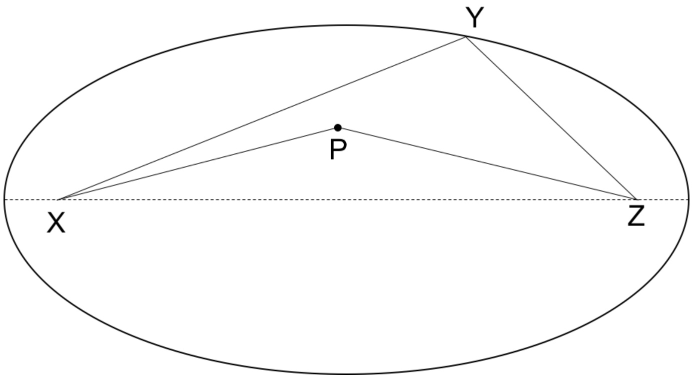

Lemma 7. When optimal branching angle solutions (Eq. (1)) result in a case where the triangle condition ($\widehat{V_jV_iV_k} \geq \theta_i$) fails, then the vertex associated with θ_i for which the inequality fails also provides the minimum of H.

Proof: Without loss of generality, let us assume that the optimal solution of θ_0 is less than the angle $\widehat{V_1 V_0 V_2}$. As ${h_0}^2 = {h_1}^2 + {h_2}^2 - 2h_1 h_2 \cos(\pi - \theta_0)$, we can form a triangle OAB with side-lengths h_0, h_1, h_2 that has the angle $\pi - \theta_0$ at the vertex A (Fig A4). Now, let us construct a triangle ABC similar to the triangle $V_0 V_1 V_2$. Drawing a line segment AC of length $h_2 \frac{v_1}{v_2}$, so that the angle $\widehat{CAB}$ equals $\widehat{V_0} \coloneqq \widehat{V_2 V_0 V_1}$, yields such a triangle with similarity ratio $\frac{h_2}{v_2}$. Hence, the side BC has length $h_2 \frac{v_0}{v_2}$ (Fig A4). Then, the side inequality applied to the concave quadrilateral OBCA (Lemma 6) leads to $h_0 + h_2 \frac{v_0}{v_2} > h_1 + h_2 \frac{v_1}{v_2}$. Multiplying both sides by v_2 provides $H_{V_1} = h_0 v_2 + h_2 v_0 > h_2 v_1 + h_1 v_2 = H_{V_0}$. In a similar manner, we can show that $H_{V_2} > H_{V_0}$, demonstrating that V_0 gives the optimal position for *J*. By symmetry, when $\theta_1 < \widehat{V_0 V_1 V_2}$ this implies the branching junction *J* collapses to V_1, and when $\theta_2 < \widehat{V_1 V_2 V_0}$, this implies that *J* collapses to V_2.

Fig A4. The diagram of the proof to show showing that when $\theta < \widehat{V_0}$, the branching junction J will collapse on V_0.

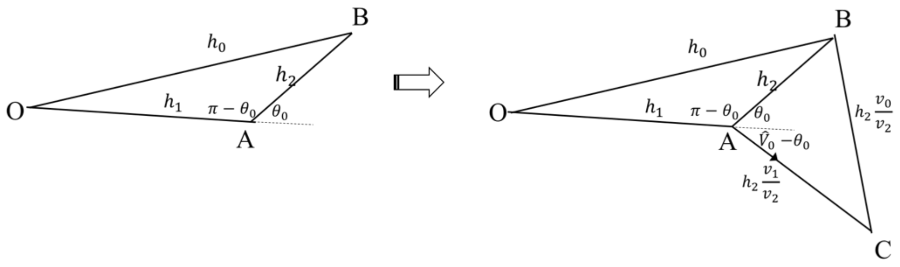

Appendix D. Power Cost Optimization for a Single Branching Junction Solutions

Here, we show that power cost optimization always leads to degenerate branching geometry. To do this, we first calculate the equivalent impedances when the branching junction *J* occurs at the vertex V_i (Fig A1)—denoted by Z_{V_i}—for each *i* .

$$Z_{V_0} = \left(\frac{1}{h_1 v_2} + \frac{1}{h_2 v_1}\right)^{-1}, \qquad Z_{V_1} = h_0 v_2 \,, \qquad Z_{V_2} = h_0 v_1$$

Now, if we show that $Z_{eq} \geq \min\left(Z_{V_0}, Z_{V_1}, Z_{V_2}\right)$, it follows that Z_{eq} attains its minimum at one of the vertices. Without loss of generality, we assume that $v_1 \leq v_2$, so $Z_{V_2} \leq Z_{V_1}$ and $\min\left(Z_{V_0}, Z_{V_1}, Z_{V_2}\right) = \min\left(Z_{V_0}, Z_{V_2}\right)$. The following lemmas verify our claim that one of the vertices is always optimal for the branching junction.

Lemma 8. Let $Z_{V_0} < Z_{V_2}$. Then, $\min\left(Z_{eq}\right) = Z_{V_0}$

Proof: To prove the lemma, we need to show that $Z_{eq} \geq Z_{V_0}$ for all possible locations of the branching junction, *J.* Because $Z_{V_0} < Z_{V_2}$, we have $h_0 > \frac{1}{v_1}\left(\frac{1}{h_1 v_2} + \frac{1}{h_2 v_1}\right)^{-1}$, so we can form the following inequality by replacing h_0 by $\left(\frac{1}{h_1 v_2} + \frac{1}{h_2 v_1}\right)^{-1} \frac{1}{v_1}$

$$Z_{eq} = h_0 l_0 + \left(\frac{1}{h_1 l_1} + \frac{1}{h_2 l_2}\right)^{-1} > \left(\frac{1}{h_1 v_2} + \frac{1}{h_2 v_1}\right)^{-1} \frac{l_0}{v_1} + \left(\frac{1}{h_1 l_1} + \frac{1}{h_2 l_2}\right)^{-1}$$

To prove $Z_{eq} \geq Z_{V_0} = \left(\frac{1}{h_1 v_2} + \frac{1}{h_2 v_1}\right)^{-1}$, it suffices to prove

$$\left(\frac{1}{h_1 v_2} + \frac{1}{h_2 v_1}\right)^{-1} \frac{l_0}{v_1} + \left(\frac{1}{h_1 l_1} + \frac{1}{h_2 l_2}\right)^{-1} \geq \left(\frac{1}{h_1 v_2} + \frac{1}{h_2 v_1}\right)^{-1}$$

Rearranging terms, the proof of the Lemma boils down to proving the inequality

$$\left(\frac{1}{h_1 l_1}+\frac{1}{h_2 l_2}\right)^{-1} > \left(1-\frac{l_0}{v_1}\right)\left(\frac{1}{h_1 v_2}+\frac{1}{h_2 v_1}\right)^{-1}, \qquad \text{(A5)}$$

Taking the reciprocal of both sides of (A5) and factoring out the terms with $\frac{1}{h_1}$ and $\frac{1}{h_2}$, this inequality is equivalent to

$$\frac{1}{h_1}\left(\frac{1}{l_1}-\frac{1}{v_2}\left(1-\frac{l_0}{v_1}\right)^{-1}\right)+\frac{1}{h_2}\left(\frac{1}{l_2}-\frac{1}{v_1}\left(1-\frac{l_0}{v_1}\right)^{-1}\right)<0$$

Hence, if we show that both of the terms in the above expression are negative, then their sum would also be negative, and the proof will be complete. In other words, it suffices to show two inequalities

$$\frac{1}{l_1}-\frac{1}{v_2}\left(1-\frac{l_0}{v_1}\right)^{-1}<0 \qquad \text{(A6)}$$

$$\frac{1}{l_2}-\frac{1}{v_1}\left(1-\frac{l_0}{v_1}\right)^{-1}<0 \qquad \text{(A7)}$$

Observe that the triangle inequality applied to the triangle V_0JV_1 gives $l_0+l_1>v_2$, hence $\frac{l_1}{v_2}>1-\frac{l_0}{v_2}>1-\frac{l_0}{v_1}$, proving (A6). Moreover, the triangle inequality applied to the triangle V_0JV_2 yields $l_0+l_2>v_1$, implying that $\frac{l_2}{v_1}>1-\frac{l_0}{v_1}$, which proves (A7). ■

The next lemma takes care of the complementary case.

Lemma 9: Let $Z_{V_0}>Z_{V_2}$, then $\min Z_{eq}=Z_{V_2}$

Proof: Following the same idea as in the proof of Lemma 8, we want to show that $Z_{eq}\geq Z_{V_2}$, or equivalently

$$\left(\frac{1}{h_1 l_1}+\frac{1}{h_2 l_2}\right)^{-1} > h_0(v_1 - l_0)$$

By the inequality (A5), we proved in Lemma 1, we have

$$\left(\frac{1}{h_1 l_1}+\frac{1}{h_2 l_2}\right)^{-1} > \left(1-\frac{l_0}{v_1}\right)\left(\frac{1}{h_1 c}+\frac{1}{h_2 v_1}\right)^{-1}$$

The assumption $\left(\frac{1}{h_1 v_2}+\frac{1}{h_2 v_1}\right)^{-1} > h_0 v_1$ further yields that

$$\left(\frac{1}{h_1 l_1}+\frac{1}{h_2 l_2}\right)^{-1} > \left(1-\frac{l_0}{v_1}\right)h_0 v_1 = h_0(v_1 - l_0)$$

as desired. ■

With Lemmas 8 and 9, we proved that the branching junction collapses onto one of the vertices for any choice of cost parameters $h_0, h_1,$ and h_2 .

Appendix E. Enlarged Consideration of the Power Cost Optimization to Go Beyond a Single Branching

In this section, we add terms c_1 and c_2 to the calculation of $\tilde{Z}_{eq}$ to respectively represent the impedance of all of the vessels are downstream from each daughter vessel at that branching junction. Furthermore, we consider the special case that impedance matching—the impedance of the parent vessel is matched by the equivalent impedances of the daughter vessels—is satisfied throughout the whole network. By requiring that siblings have identical impedances and that each sibling has the same number of downstream vessels, we show that the ratio $\frac{c_i}{Z_i}$ is larger for vessels that are near to the first branching level (i.e., the heart). To simplify the calculations, we

enumerate the levels such that the level number increases from capillary (level 0) to the heart (level N). This is the reverse of the labeling used in most models.

By applying impedance matching successively from level 0 to level *k*, we first recognize that the impedance of the vessel at the kth level is given by $Z_0/2^k$, where Z_0 denotes the impedance of the capillary. Moreover, for the first few levels above the capillary level (when $k = 0, 1, 2$), we find that the downstream impedance at level *k* follows the form $\frac{kZ}{2^k}$ (Fig A5). The next Lemma generalizes this formula for all levels *k*.

Lemma 10. The downstream impedance from a daughter vessel at level *k* is given by

$$c_k = \frac{kZ_0}{2^k}$$

Proof: We prove this claim by induction. Note that a vessel at level $k-1$ is in series with the downstream vessels as illustrated in the Fig A5. If the downstream impedance at level $(k-1)$ is equal to $\frac{(k-1)Z}{2^{k-1}}$, then by rules of fluid mechanics, the downstream impedance at level *k* is given by

$$c_k = \frac{1}{\frac{1}{\frac{Z_0}{2^{k-1}} + \frac{(k-1)Z_0}{2^{k-1}}} + \frac{1}{\frac{Z_0}{2^{k-1}} + \frac{(k-1)Z_0}{2^{k-1}}}} = \frac{kZ_0}{2^k}. \blacksquare$$

Hence, by Lemma 9, we have that the value of c_k/Z_k at level k is equal to

$$\frac{\frac{kZ_0}{2^k}}{\frac{Z_0}{2^k}} = k$$

so that the value of this ratio increases with the level (i.e., increase from the capillaries to the heart). Therefore, near the heart, the constants (c_i) representing the downstream impedances in the optimization scheme are relatively large compared to the impedances (Z_i) of the daughter vessels at that branching junction.

Figure A5. (a) Perfectly-balanced branching network with identical daughter impedances and **(b)** inclusion of impedances for downstream vessels in entire branching network and thus beyond just the branching level *k*.

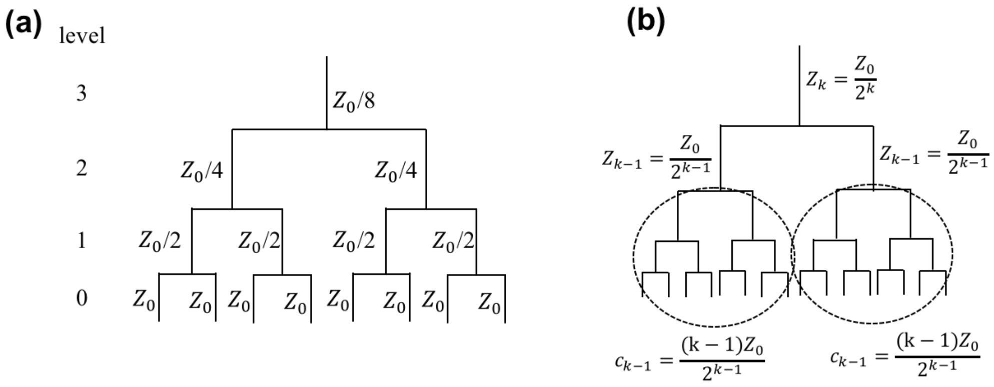

References

1. Zamir M. Nonsymmetrical bifurcations in arterial branching. The Journal of general physiology. 1978;72(6):837-45.

2. Zamir M. Optimality principles in arterial branching. Journal of Theoretical Biology. 1976;62(1):227-51.

3. Murray CD. The physiological principle of minimum work applied to the angle of branching of arteries. The Journal of general physiology. 1926;9(6):835-41.
