# Supplementary material for: Do Vascular Networks Branch Optimally or Randomly across Spatial Scales?
Source: PLoS Comput Biol. 2016 Nov 30;12(11):e1005223. doi: 10.1371/journal.pcbi.1005223 (PMC5130167; doi:10.1371/journal.pcbi.1005223)

S1 Fig. Planarity of local branching junctions in mouse and human networks. When the parent and child vessels are colocalized in the same plane, the junction is considered planar. **(a)** We quantify the planarity measure of local branching is by the scaled distance formula given here. When the scaled distance is equal to 0, the branching is perfectly planar. Larger values of the scaled distance formula imply stronger deviations from planarity. **(b-c)** Histograms of the scaled distance for (b) the mouse lung and (c) the human head and torso are skewed towards the perfectly planar case, providing evidence that the local branching is mostly planar for both networks.

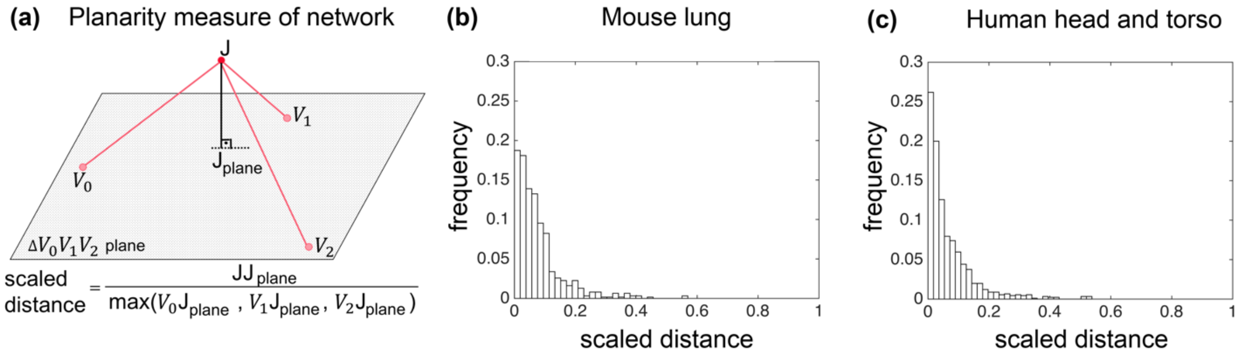

Supplement: S1 Fig — When the parent and child vessels are colocalized in the same plane, the junction is considered planar. (a) We quantify the planarity measure of local branching is by the scaled distance formula given here. When the scaled distance is equal to 0, the branching is perfectly planar. Larger values of the scaled distance formula imply stronger deviations from planarity. (b-c) Histograms of the scaled distance for (b) the mouse lung and (c) the human head and torso are skewed towards the perfectly planar case, providing evidence that the local branching is mostly planar for both networks. (PDF) [file pcbi.1005223.s002.pdf]
