# Supplementary material for: Do Vascular Networks Branch Optimally or Randomly across Spatial Scales?
Source: PLoS Comput Biol. 2016 Nov 30;12(11):e1005223. doi: 10.1371/journal.pcbi.1005223 (PMC5130167; doi:10.1371/journal.pcbi.1005223)

S2 Fig. Type of optimal branching solution and analysis of Murray's Law. Non-degenerate solutions (branching occurs) are specified with white, and degenerate solutions (junction collapses to vessel endpoint so no branching) are specified with black. The red color represents the region where the Generalized Murray's law holds (scaling exponent $d \in [2,3]$), which is a subset of the region of non-degenerate region. **(a)** Material cost: Surface-area, **(b)** Material cost: Volume, **(c)** Analysis of vasculature for mouse lung shows that the Generalized Murray's Law hold only for 10% (=66/633) of the branching data, **(d)** Analysis of human head and torso shows that the Generalized Murray's Law holds only for 13% (=112/849) of branching data.

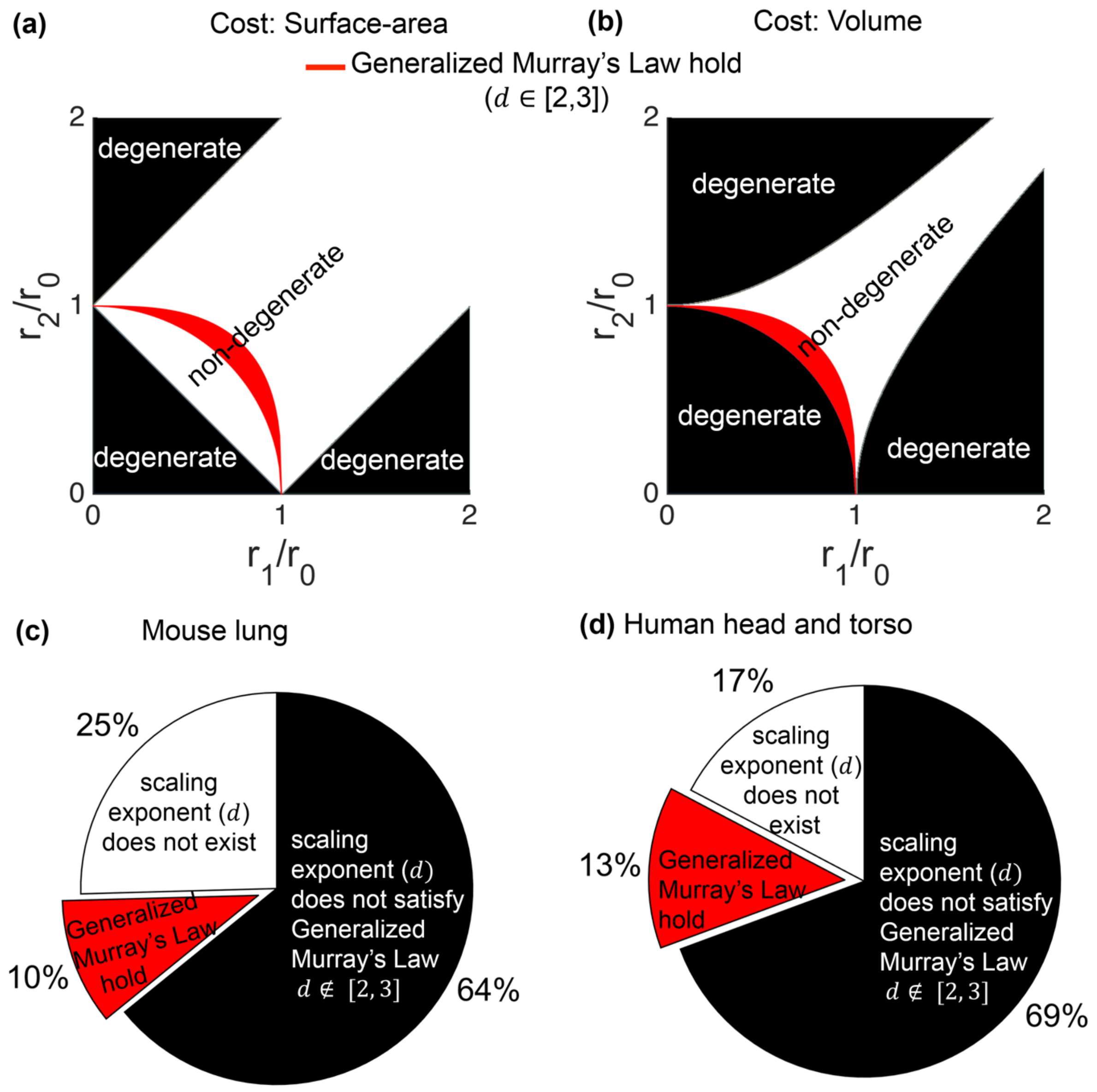

Supplement: S2 Fig — Non-degenerate solutions (branching occurs) are specified with white, and degenerate solutions (junction collapses to vessel endpoint so no branching) are specified with black. The red color represents the region where the Generalized Murray’s law holds (scaling exponent d ∈ [2,3]), which is a subset of the region of non-degenerate region. (a) Material cost: Surface-area, (b) Material cost: Volume, (c) Analysis of vasculature for mouse lung shows that the Generalized Murray’s Law hold only for 10% (= 66/633) of the branching data, (d) Analysis of human head and torso shows that the Generalized Murray’s Law holds only for 13% (= 122/914) of branching data. (PDF) [file pcbi.1005223.s003.pdf]
