# Supplementary material for: Do Vascular Networks Branch Optimally or Randomly across Spatial Scales?
Source: PLoS Comput Biol. 2016 Nov 30;12(11):e1005223. doi: 10.1371/journal.pcbi.1005223 (PMC5130167; doi:10.1371/journal.pcbi.1005223)

S3 Fig. Heat map (color map) representation of the equivalent impedance (i.e., Z_{eq}) for PC-0 (power-cost optimization for a single branching junction). The optimal branching location, J, for different choice of cost parameters (h_i) and end points (V_i) is marked with a green dot. The optimal branching junction coincides with the end point V_0 in panel (a), V_1 in panel (b), and V_2 in panel (c). This highlights the fact that, depending on both the vessel cost and the geometry of the endpoints, the branching junction can collapse on the parent or either of the daughter vessel endpoints.

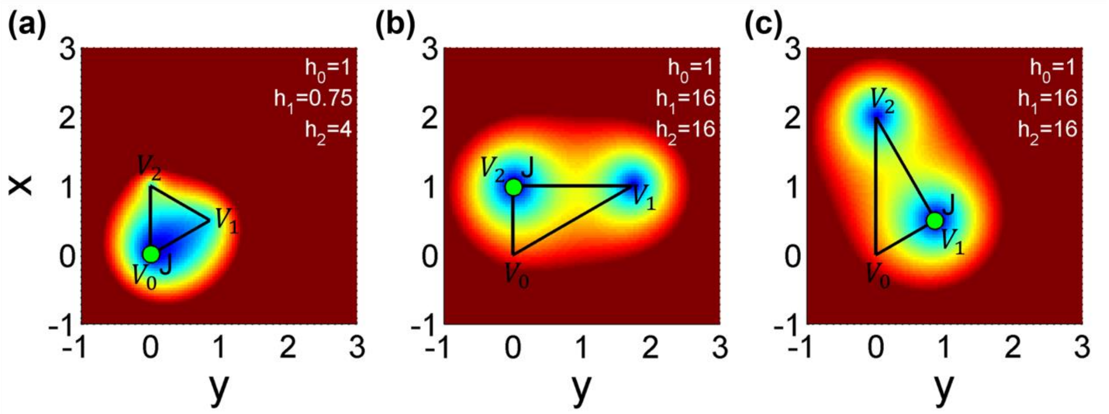

Supplement: S3 Fig — The optimal branching location, J, for different choice of cost parameters (hi) and end points (Vi) is marked with a green dot. The optimal branching junction coincides with the end point V0 in panel (a), V1 in panel (b), and V2 in panel (c). This highlights the fact that, depending on both the vessel cost and the geometry of the endpoints, the branching junction can collapse on the parent or either of the daughter vessel endpoints. (PDF) [file pcbi.1005223.s004.pdf]
