# Supplementary material for: Do Vascular Networks Branch Optimally or Randomly across Spatial Scales?
Source: PLoS Comput Biol. 2016 Nov 30;12(11):e1005223. doi: 10.1371/journal.pcbi.1005223 (PMC5130167; doi:10.1371/journal.pcbi.1005223)
Supplement: S4 Fig — The vessels and the fixed end points for the real branching network are in red. The random branching simulations start from this real networks, and newly resulting branching locations and vessels are shown in black. (1) The first simulation starts by randomly positioning the most upstream branching junction of the network (green) within the corresponding triangle of end points (green dashed triangle). The simulation iteratively continues this process by updating the new branching junction and determining the branching junctions at the next level (blue and purple) in the same way. Note that this first intermediate constraint places the branching point onto the plane of the current vessel end points, hence constraining all three vessels at a branching junction to be confined to a single plane. (2) The second simulation starts by determining the locations of the most downstream branching junctions (blue and purple) of the network. The first iteration is to randomly position new branching junctions within a spherical boundary (blue and purple dashed spheres) whose size is proportional to the distance between the two downstream endpoints (i.e., terminal tips) of the current branching junction. The simulation then iteratively builds backwards by randomly placing branching junctions within corresponding spherical boundaries at each branching generation until the first branching node is reached. These spherical boundaries relax the assumption of local planarity and lead to branching at the local level for which the three vessels connected at a branching junction do not need to be co-planar. (PDF) [file pcbi.1005223.s005.pdf]

S4 Fig. Iterations for randomly branching networks with intermediate constraints. The vessels and the fixed end points for the real branching network are in red. The random branching simulations start from this real networks, and newly resulting branching locations and vessels are shown in black. (1) The first simulation starts by randomly positioning the most upstream branching junction of the network (green) within the corresponding triangle of end points (green dashed triangle). The simulation iteratively continues this process by updating the new branching junction and determining the branching junctions at the next level (blue and purple) in the same way. Note that this first intermediate constraint places the branching point onto the plane of the current vessel end points, hence constraining all three vessels at a branching junction to be confined to a single plane. (2) The second simulation starts by determining the locations of the most downstream branching junctions (blue and purple) of the network. The first iteration is to randomly position new branching junctions within a spherical boundary (blue and purple dashed spheres) whose size is proportional to the distance between the two downstream endpoints (i.e., terminal tips) of the current branching junction. The simulation then iteratively builds backwards by randomly placing branching junctions within corresponding spherical boundaries at each branching generation until the first branching node is reached. These spherical boundaries relax the assumption of local planarity and lead to branching at the local level for which the three vessels connected at a branching junction do not need to be co-planar.

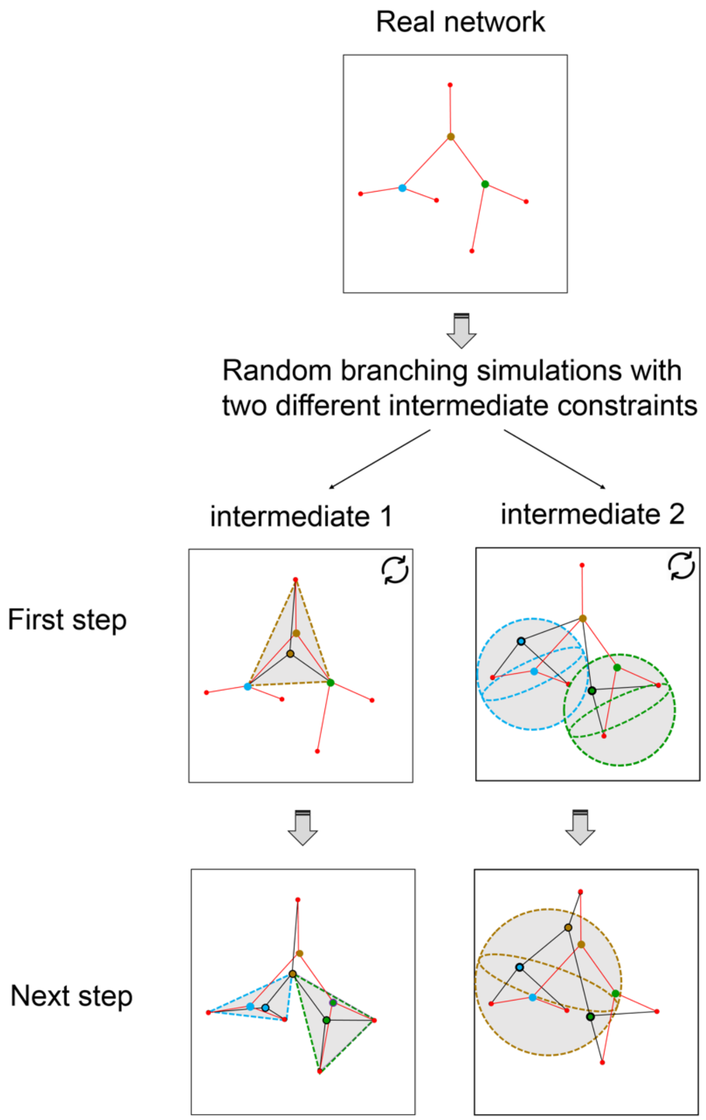
Real network
Random branching simulations with
two different intermediate constraints
intermediate 1
intermediate 2
First step
Next step
