# Supplementary material for: Do Vascular Networks Branch Optimally or Randomly across Spatial Scales?
Source: PLoS Comput Biol. 2016 Nov 30;12(11):e1005223. doi: 10.1371/journal.pcbi.1005223 (PMC5130167; doi:10.1371/journal.pcbi.1005223)

S5 Fig. Histogram of branching angles and optimal branching angles of MC (material-cost) optimizations for mouse and human networks. All histograms show a unimodal distribution with means given by the red vertical lines.

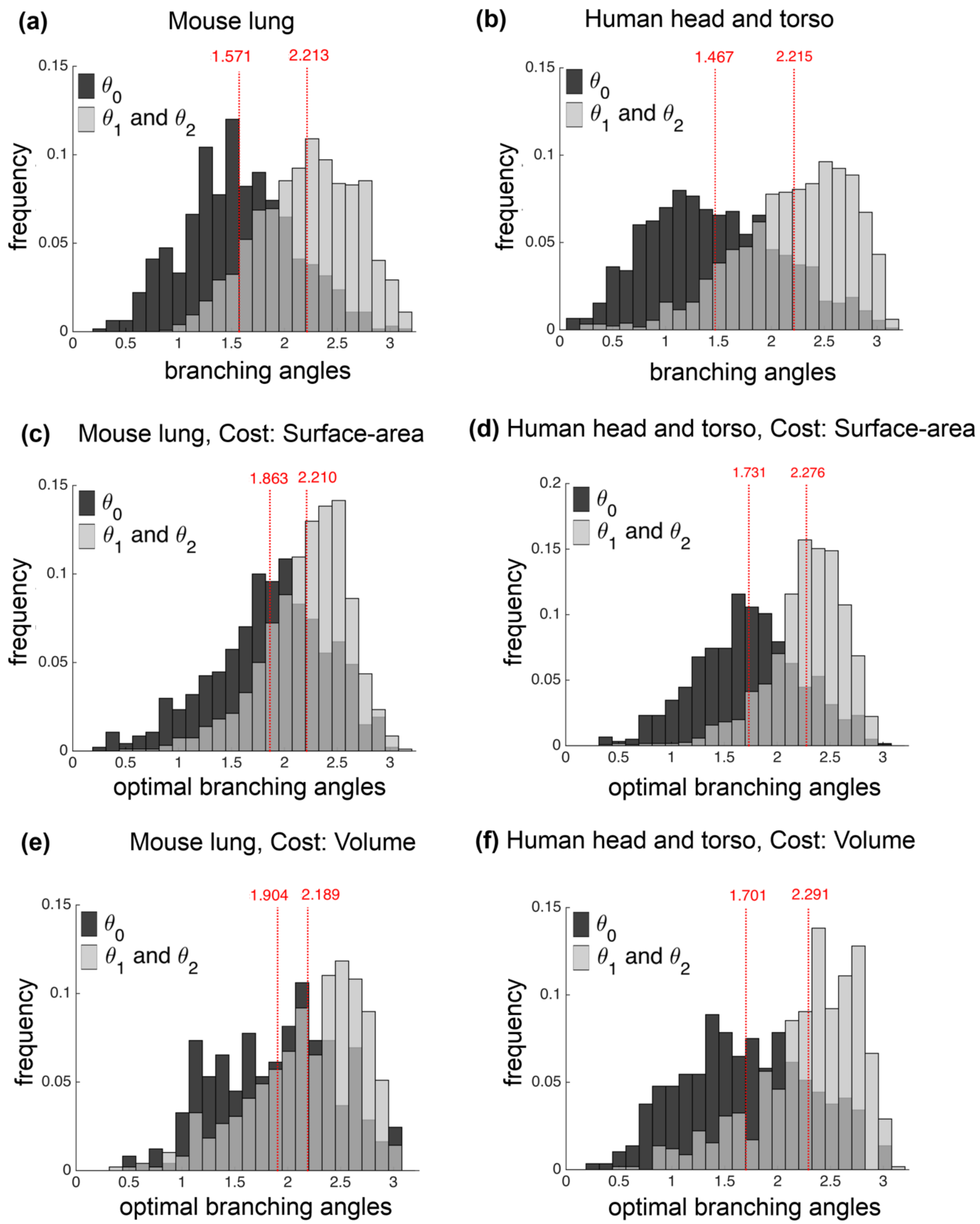

Supplement: S5 Fig — All histograms show a unimodal distribution with means given by the red vertical lines. (PDF) [file pcbi.1005223.s006.pdf]
