# Supplementary material for: Do Vascular Networks Branch Optimally or Randomly across Spatial Scales?
Source: PLoS Comput Biol. 2016 Nov 30;12(11):e1005223. doi: 10.1371/journal.pcbi.1005223 (PMC5130167; doi:10.1371/journal.pcbi.1005223)

S6 Fig. Fraction of degenerate and non-degenerate branching solutions for material-cost optimizations. The surface-area constraint leads to degenerate solutions for **(a)** 26% (=163/633) of branching junctions in the mouse lung network and **(b)** 34% (=292/849) of branching junctions for the human head and torso network. Volume constraint leads to degenerate solutions for **(c)** 61% (=388/633) of branching junctions in the mouse lung network and **(d)** 68% (576=/849) of branching junctions for the human head and torso network.

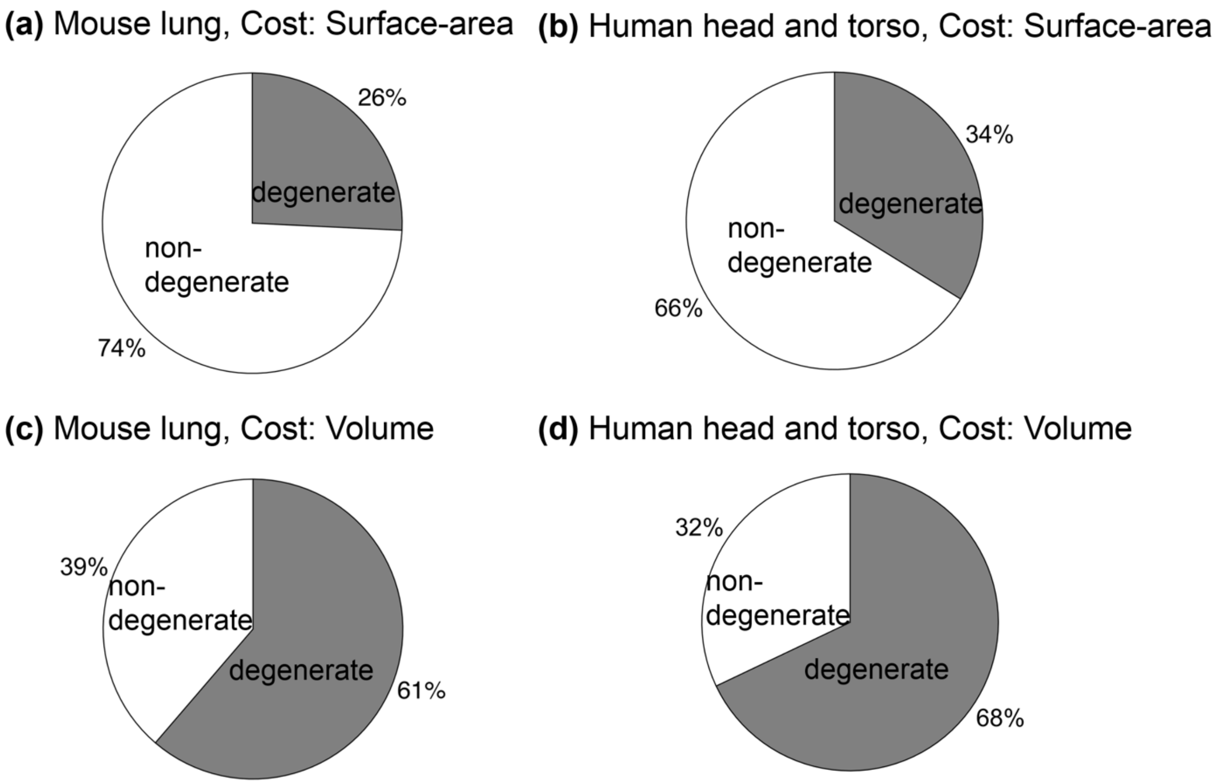

Supplement: S6 Fig — The surface-area constraint leads to degenerate solutions for (a) 26% (= 163/633) of branching junctions in the mouse lung network and (b) 34% (= 309/914) of branching junctions for the human head and torso network. Volume constraint leads to degenerate solutions for (c) 61% (= 388/633) of branching junctions in the mouse lung network and (d) 68% (= 621/914) of branching junctions for the human head and torso network. (PDF) [file pcbi.1005223.s007.pdf]
