# Supplementary material for: Do Vascular Networks Branch Optimally or Randomly across Spatial Scales?
Source: PLoS Comput Biol. 2016 Nov 30;12(11):e1005223. doi: 10.1371/journal.pcbi.1005223 (PMC5130167; doi:10.1371/journal.pcbi.1005223)

S7 Fig. Junction-level comparison of optimal versus actual branching angles for the surface-area constraint of material-cost optimizations. Results for **(a)** mouse lung and **(b)** human head and torso. The Pearson correlation coefficients are calculated for each plot.

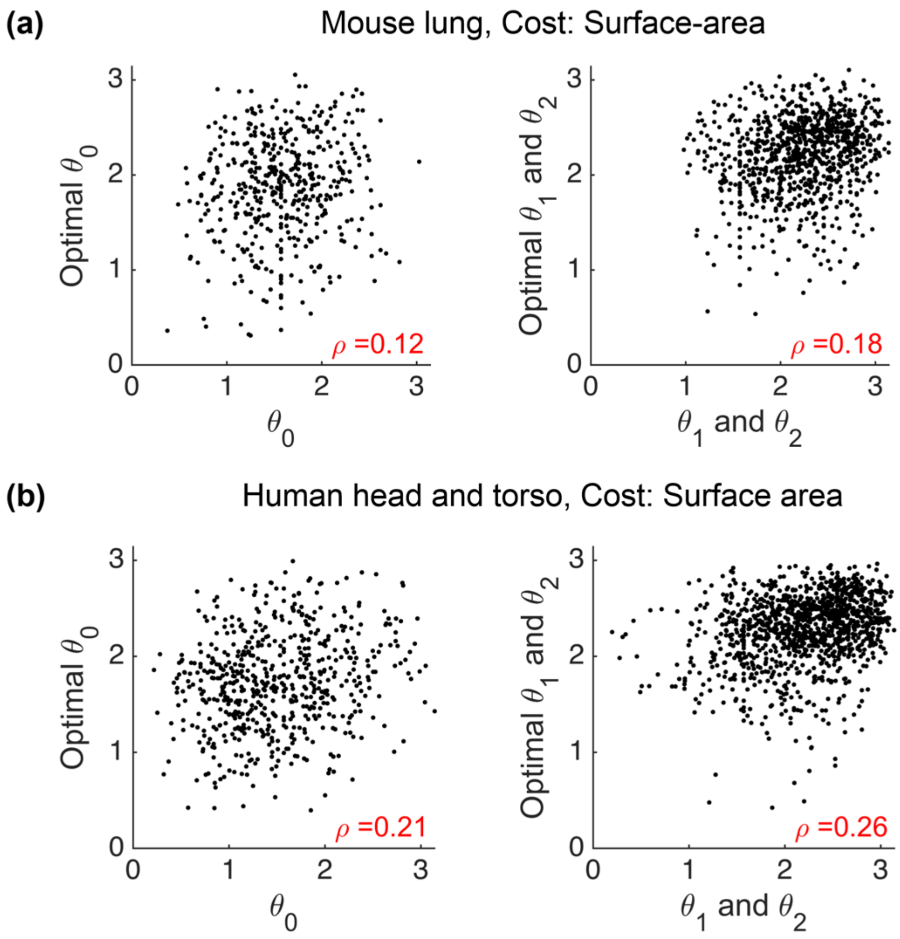

Supplement: S7 Fig — Results for (a) mouse lung and (b) human head and torso. The Pearson correlation coefficients are calculated for each plot. (PDF) [file pcbi.1005223.s008.pdf]
