# Supplementary material for: Do Vascular Networks Branch Optimally or Randomly across Spatial Scales?
Source: PLoS Comput Biol. 2016 Nov 30;12(11):e1005223. doi: 10.1371/journal.pcbi.1005223 (PMC5130167; doi:10.1371/journal.pcbi.1005223)
Supplement: S8 Fig — The real and simulated networks (via local to global constraints) are separated by different columns. A schematic small network is given to describe how different simulations are performed. The vessels and the fixed end points of the real branching network are represented in red. Vessels that result from random branching simulations are in black. The real human head and torso network and the simulated human head and torso networks are shown within a minimum spherical boundary that contains all branching data from the real network. Here, the red nodes for each figure correspond to the real data, whereas the black nodes correspond to the simulated data. Note that the terminal tips and the most upstream node (i.e., the source) are determined from real data and fixed throughout all simulations. The resulting asymmetry ratio distributions for length and branching angles are provided for the real network and for each of the simulations. The statistical comparisons of random branching simulations with empirical data are given in Table 1. (PDF) [file pcbi.1005223.s009.pdf]

S8 Fig. Comparison of real data for vascular networks versus random simulations of branching junctions for human head and torso network. The real and simulated networks (via local to global constraints) are separated by different columns. A schematic small network is given to describe how different simulations are performed. The vessels and the fixed end points of the real branching network are represented in red. Vessels that result from random branching simulations are in black. The real human head and torso network and the simulated human head and torso networks are shown within a minimum spherical boundary that contains all branching data from the real network. Here, the red nodes for each figure correspond to the real data, whereas the black nodes correspond to the simulated data. Note that the terminal tips and the most upstream node (i.e., the source) are determined from real data and fixed throughout all simulations. The resulting asymmetry ratio distributions for length and branching angles are provided for the real network and for each of the simulations. The statistical comparisons of random branching simulations with empirical data are given in Table 1.

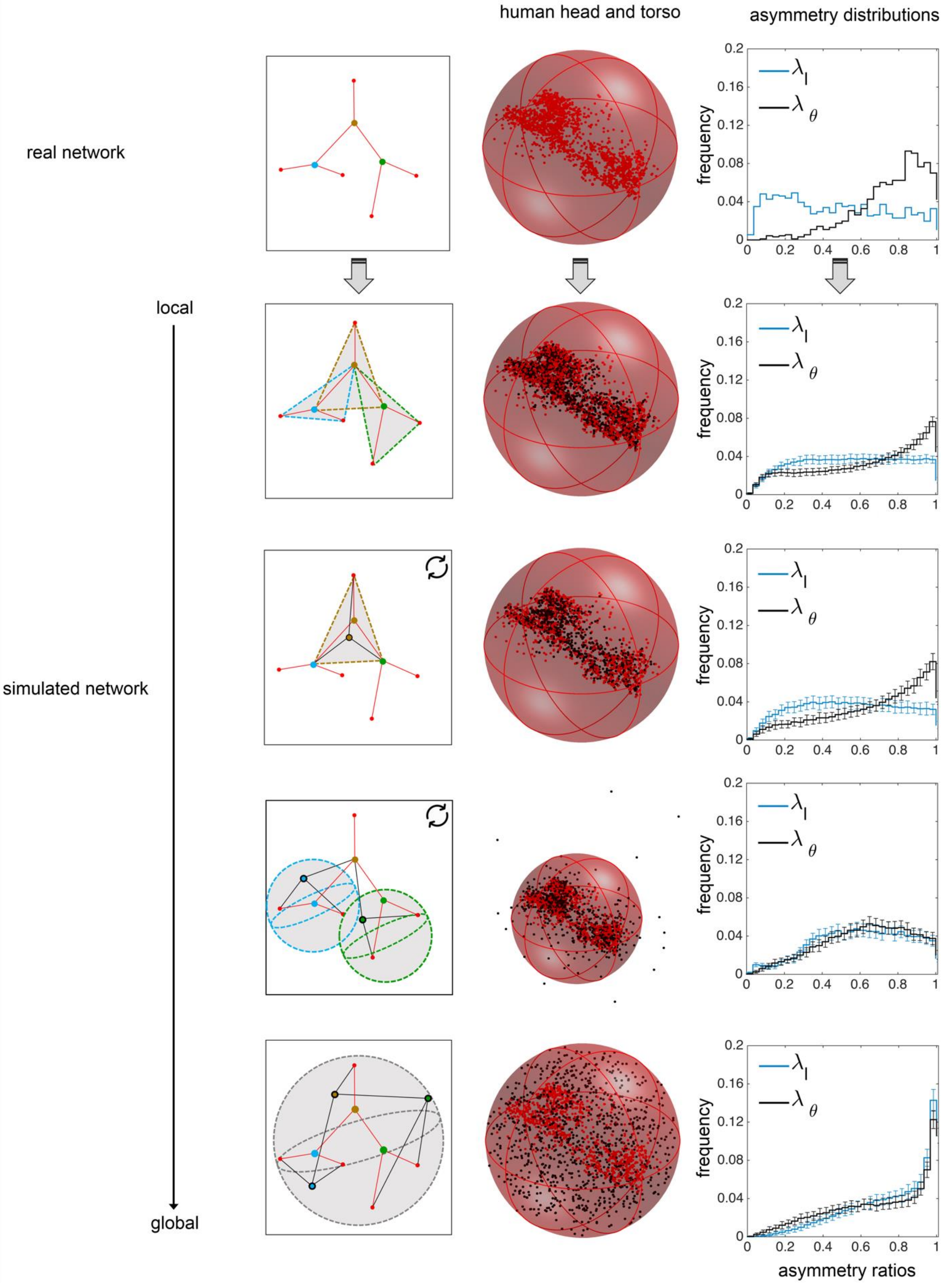

human head and torso
asymmetry distributions
real network
local
simulated network
global
frequency
asymmetry ratios
λ_l
λ_θ
