# Supplementary material for: Do Vascular Networks Branch Optimally or Randomly across Spatial Scales?
Source: PLoS Comput Biol. 2016 Nov 30;12(11):e1005223. doi: 10.1371/journal.pcbi.1005223 (PMC5130167; doi:10.1371/journal.pcbi.1005223)

S9 Fig. Computing branching angles from extracted vessel skeleton. We calculate branching angles as angles between the straight lines defined by the positional coordinates of the vessels—the endpoint coordinates V_0, V_1, V_2, and J—at the bifurcation.

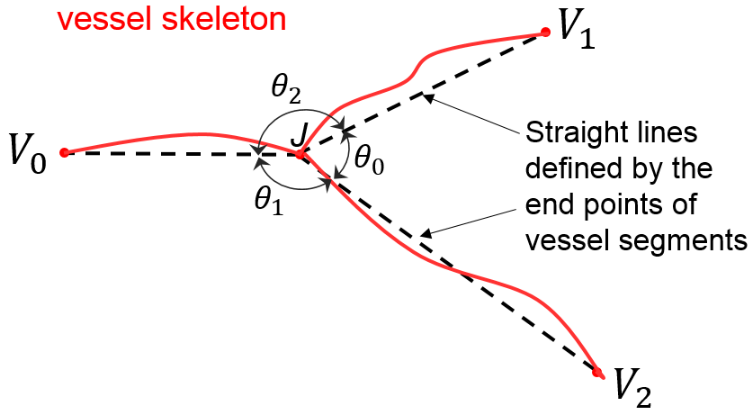

Supplement: S9 Fig — We calculate branching angles as angles between the straight lines defined by the positional coordinates of the vessels—the endpoint coordinates V0, V1, V2, and J—at the bifurcation. (PDF) [file pcbi.1005223.s010.pdf]
