# Supplementary material for: Do Vascular Networks Branch Optimally or Randomly across Spatial Scales?
Source: PLoS Comput Biol. 2016 Nov 30;12(11):e1005223. doi: 10.1371/journal.pcbi.1005223 (PMC5130167; doi:10.1371/journal.pcbi.1005223)
Supplement: S1 Table — (PDF) [file pcbi.1005223.s011.pdf]

	Newberry et al.	this study
Angicart GitHub version	83df119	ab81935
human subject 1	0.28	0.30
human subject 2	0.34	0.35
human subject 3	0.68	0.70
human subject 4	0.65	0.65
human subject 5	0.76	0.75
human subject 6	0.88	0.90
human subject 7	0.40	0.40
human subject 8	0.63	0.65
human subject 9	0.65	0.65
human subject 10	0.78	0.80
human subject 11	0.70	0.70
human subject 12	0.31	0.30
human subject 13	0.67	0.65
human subject 14	0.70	0.70
human subject 15	0.64	0.65
human subject 16	0.34	0.35
human subject 17	0.50	0.50
human subject 18	0.51	0.50
mouse lung	N/A	0.90
