# Supplementary material for: Do Vascular Networks Branch Optimally or Randomly across Spatial Scales?
Source: PLoS Comput Biol. 2016 Nov 30;12(11):e1005223. doi: 10.1371/journal.pcbi.1005223 (PMC5130167; doi:10.1371/journal.pcbi.1005223)
Supplement: S2 Table — A vessel is identified as non-deformed when the number of voxels outside a distance rad+1 from the centerline of the vessel is less than the 10% of the total voxels of the vessel segment [29, 43]. (PDF) [file pcbi.1005223.s012.pdf]

	% of non-deformed vessels
human head and torso (Newberry et al.)	41
human head and torso (this study)	58
mouse lung (this study)	88
