# Supplementary material for: Do Vascular Networks Branch Optimally or Randomly across Spatial Scales?
Source: PLoS Comput Biol. 2016 Nov 30;12(11):e1005223. doi: 10.1371/journal.pcbi.1005223 (PMC5130167; doi:10.1371/journal.pcbi.1005223)
Supplement: S3 Table — (PDF) [file pcbi.1005223.s013.pdf]

Data	Cost	Mean±SD	
		θ_0	θ_1 and θ_2
Combined data	N/A (Real data)	1.510±0.583	2.214±0.512
	Surface-area	1.789±0.518	2.247±0.385
	Volume	1.793±0.590	2.445±0.510
Mouse lung	N/A (Real data)	1.571±0.513	2.213±0.460
	Surface-area	1.863±0.536	2.210±0.406
	Volume	1.904±0.566	2.189±0.521
Human head and torso	N/A (Real data)	1.467±0.624	2.215±0.546
	Surface-area	1.731±0.497	2.276±0.366
	Volume	1.701±0.594	2.291±0.495
