# Supplementary material for: Do Vascular Networks Branch Optimally or Randomly across Spatial Scales?
Source: PLoS Comput Biol. 2016 Nov 30;12(11):e1005223. doi: 10.1371/journal.pcbi.1005223 (PMC5130167; doi:10.1371/journal.pcbi.1005223)
Supplement: S1 Text — (PDF) [file pcbi.1005223.s014.pdf]

S1 Text. Details on the empirical data and the choice of parameters

As explained in the Materials and Methods, we first preprocessed the images to acquire high quality data and to reduce the computation time. In Newberry et al. [1], the noisy data has been further treated by filtering out the vessels that are possibly misclassified by Angicart. However, this is not applicable in our study for two reasons. First, because the focus of our study is on the branching geometries, excluding one vessel usually eliminates two branching junctions, hence having a much larger effect on the size of the overall dataset for branching junctions and asymmetry ratios. Second, eliminating an intermediate-size vessel disconnects the network, so our simulations for testing the random branching hypothesis (see main text: Exploring from local to global constraints for the random placement of branching junctions) would be inaccurate or incomplete. For these two reasons, we need to acquire as high quality data as possible from the first step of extraction from the image. For this reason, we use strict intensity thresholds for each dataset and a version of Angicart that is less inclusive of blurry vessels or tips in order to produce the network data. The exact thresholds and version of Angicart are given in S1 Table. Note that threshold values and the version of Angicart differ from those in Newberry et al. [1], which tried to be as inclusive of vessel data as possible and resulted in a lower percentage of high-quality, non-deformed vessels (S2 Table). Consequently, performing the same analysis in our paper on the Newberry et al. dataset will give slightly different results. For completeness, we also performed our statistical comparison analysis of optimal and random branching patterns to the human head and torso data published in [1]. We find that the first few moments reveal similar conclusions as in the Table 1, whereas none of the models yield significant KL distance p-values.

References

1. Newberry MG, Ennis DB, Savage VM. Testing Foundations of Biological Scaling Theory Using Automated Measurements of Vascular Networks. PLoS Comput Biol. 2015;11(8):e1004455.
