# Supplementary material for: Do Vascular Networks Branch Optimally or Randomly across Spatial Scales?
Source: PLoS Comput Biol. 2016 Nov 30;12(11):e1005223. doi: 10.1371/journal.pcbi.1005223 (PMC5130167; doi:10.1371/journal.pcbi.1005223)
Supplement: S2 Text — (PDF) [file pcbi.1005223.s015.pdf]

S2 Text. Murray's law and optimal branching angle solutions

Previous work on the branching angles [1-4] presumes Murray's law holds for vessel radii. Murray's Law relates the parent vessel radius (r_p) to its daughter vessel radii (r_d) via the formula $r_p{}^3 = \sum r_d{}^3$ [5]. Murray's law is derived based on two assumptions: (i) local conservation of fluid at a branching junction and (ii) joint minimization of the sum of the power loss of blood flow and the power cost of the blood volume for a single vessel.

Based on analysis of some vascular data [4, 6, 7], the scaling exponent, $d,$ relating the radii of vessels at a single branching junction is assumed to be within the range 2 to 3. For a bifurcation the generalized version of the Murray's law is given by

$$r_0{}^d = r_1{}^d + r_2{}^d$$

where the subscripts 0,1 and 2 denote the parent and the two daughter vessels respectively (Fig 1c). Here, we show that when Murray's law holds, the branching angle solution for *material-cost (MC) optimizations* (see Main Text) yields non-degenerate branching geometries. That is, the branching junction is not located at one of the endpoints of the vessels, meaning that a branching actually does occur.

Recall that we represent the material cost optimizations in the generic form of a cost function as $H = \sum h_i l_i$, where the cost per length for a given vessel is denoted by $h_i \coloneqq r_i^k$. Here, k is defined based on the specific optimization constraint such that $k = 1$ or 2 for the surface-area and the volume optimization, respectively. Recall also that the branching solution is degenerate (i.e., the junction collapse to one of the vessel endpoints, implying no branching) if one of the inequalities $h_i \geq h_j + h_k$ for any combination of (i, j, k) is satisfied.

To illustrate the regions where the cost parameters lead to degenerate branching solutions, we first numerically analyze the space of $\frac{r_1}{r_0}$ and $\frac{r_2}{r_0}$ (S2a, b Fig). In doing so, we color the regions based on their outcomes (non-degenerate versus degenerate optimal solutions). We also specify the region where the Generalized Murray's law is satisfied. We find the Generalized Murray's Law region is a subset of the non-degenerate solution region. This means that if we focus on the radii satisfying the Generalized Murray's law, we will not observe any degenerate solutions. This explains why degeneracies are not observed or discussed in previous work on the optimal branching angles [1].

We now prove analytically that if Generalized Murray's law holds, $h_i \leq h_j + h_k$ for any combination of (i, j, k) or equivalently, the optimal branching solution leads to non-degenerate branching geometry. By the Generalized Murray's law, d is greater than 1, so the parent radius is greater than the daughter radius, i.e. $r_0 \geq r_1, r_2$. Combining this inequality with the fact that cost increases with radius (since $h_i = r_i^k$ and $k > 0$) for material cost optimizations, it follows that $h_1 \leq h_0 + h_2$ and $h_2 \leq h_0 + h_1$. Hence, it suffices to prove that $h_0 \leq h_1 + h_2$. Dividing both sides of this inequality by h_1 and substituting $h_i = r_i^k$, we want to show

$$\left(\frac{r_0}{r_1}\right)^k \leq 1 + \left(\frac{r_2}{r_1}\right)^k \qquad \text{S1.}$$

We note that the ratio of the exponents *k* and *d* ($i.e., R \coloneqq \frac{k}{d}$) is bounded between 0 and 1 ($1/3 \leq R \leq 1$). This is because $1 \leq k \leq 2$ for the area or volume constraints in material cost optimization and $3 \geq d \geq 2$ for Generalized Murray's Law. Moreover, dividing both sides of Generalized Murray's Law by r_1^d and defining $\alpha \coloneqq \left(\frac{r_2}{r_1}\right)^d$, we have

$$\left(\frac{r_0}{r_1}\right)^d = 1 + \left(\frac{r_2}{r_1}\right)^d = 1 + \alpha \qquad \text{S2.}$$

Substituting S2 into S1, the inequality S1 becomes

$$(1+\alpha)^R \leq 1 + \alpha^R$$

Now, we define the continuous function $f(\alpha) = (1+\alpha)^R - 1 - \alpha^R$ and observe that $f(0) = 0$. Taking the derivative of f, we get $f'(\alpha)/R = (1+\alpha)^{R-1} - \alpha^{R-1}$. Since $-2/3 \leq R - 1 \leq 2/3$, $(1+\alpha)^{R-1} < \alpha^{R-1}$, so $f'(\alpha)$<0. Because $f(0) = 0$, this means $f(\alpha) = (1+\alpha)^t - 1 - \alpha^t < 0$ when $\alpha > 0$, which is always the case for physically meaningful values for radius, which must be positive. Therefore, the inequality S1 and the equivalent version above, $(1+\alpha)^t \leq 1 + \alpha^t$, both hold, proving our result. ■

Because of this result, previous studies such as Zamir [1-4] always found non-degenerate solutions and did not consider the possibility of degeneracies. We have real empirical data for the radii, lengths, and branching angles for many branching junctions in human head and torso and mouse lung. In light of the above results, we input the vessel radius values for each junction and numerically solved the scaling exponent (d) to determine how often the Generalized Murray's Law holds (S2c, d Fig). For both datasets, we show that the majority of branching junctions lead to scaling exponents that do not satisfy Generalized Murray's law.

References

1. Zamir M. Nonsymmetrical bifurcations in arterial branching. The Journal of general physiology. 1978;72(6):837-45.
2. Murray CD. The physiological principle of minimum work applied to the angle of branching of arteries. The Journal of general physiology. 1926;9(6):835-41.
3. Kamiya A, Togawa T. Optimal branching structure of the vascular tree. The Bulletin of mathematical biophysics. 1972;34(4):431-8.
4. Changizi MA, Cherniak C. Modeling the large-scale geometry of human coronary arteries. Canadian journal of physiology and pharmacology. 2000;78(8):603-11.
5. Murray CD. The Physiological Principle of Minimum Work. I. The Vascular System and the Cost of Blood Volume. Proc Natl Acad Sci. 1926;12(3):207-14.
6. Dawson CA, Krenz GS, Karau KL, Haworth ST, Hanger CC, Linehan JH. Structure-function relationships in the pulmonary arterial tree. Journal of Applied Physiology. 1999;86(2):569-83.
7. Labarbera M. The design of fluid transport systems: a comparative perspective. Flow-Dependent Regulation of Vascular Function: Springer; 1995. p. 3-27.
